# Ethnic Diversity and Warfarin Pharmacogenomics

**DOI:** 10.3389/fphar.2022.866058

**Published:** 2022-04-04

**Authors:** Innocent G. Asiimwe, Munir Pirmohamed

**Affiliations:** The Wolfson Centre for Personalized Medicine, MRC Centre for Drug Safety Science, Department of Pharmacology and Therapeutics, Institute of Systems, Molecular and Integrative Biology, University of Liverpool, Liverpool, United Kingdom

**Keywords:** ancestry, ethnic diversity, underrepresented populations, pharmacogenetics, pharmacogenomics

## Abstract

Warfarin has remained the most commonly prescribed vitamin K oral anticoagulant worldwide since its approval in 1954. Dosing challenges including having a narrow therapeutic window and a wide interpatient variability in dosing requirements have contributed to making it the most studied drug in terms of genotype-phenotype relationships. However, most of these studies have been conducted in Whites or Asians which means the current pharmacogenomics evidence-base does not reflect ethnic diversity. Due to differences in minor allele frequencies of key genetic variants, studies conducted in Whites/Asians may not be applicable to underrepresented populations such as Blacks, Hispanics/Latinos, American Indians/Alaska Natives and Native Hawaiians/other Pacific Islanders. This may exacerbate health inequalities when Whites/Asians have better anticoagulation profiles due to the existence of validated pharmacogenomic dosing algorithms which fail to perform similarly in the underrepresented populations. To examine the extent to which individual races/ethnicities are represented in the existing body of pharmacogenomic evidence, we review evidence pertaining to published pharmacogenomic dosing algorithms, including clinical utility studies, cost-effectiveness studies and clinical implementation guidelines that have been published in the warfarin field.

## 1 Introduction

Warfarin, first approved in 1954 (Lim, 2017), remains the most commonly used vitamin K oral anticoagulant worldwide (Huynh, 2017). For example, in 2019, warfarin accounted for more than seven million prescriptions in England, making it the most frequently prescribed oral anticoagulant (Afzal et al., 2021). It is used in the prevention and/or treatment of thromboembolic disorders, such as venous thromboembolism, rheumatic heart disease, and prevention of strokes in patients with atrial fibrillation. Despite being used for close to 70 years, warfarin dosing remains challenging, one reason being its narrow therapeutic window. Below the minimum therapeutic dose, there is a high risk of thrombotic events due to inadequate anticoagulation (lack of efficacy) whereas bleeding becomes increasingly likely when doses exceed the maximum therapeutic dose (Lee and Klein, 2013). A narrow therapeutic window implies that the minimum and maximum therapeutic doses are close together and hence great care is required to maintain warfarin doses within the safe limits. This is complicated by high inter-patient and intra-patient variabilities in warfarin dosing requirements. For example, due to genetic and other factors, daily dosing requirements can be as low as 0.5 mg (Vanscoy and McAuley, 1991) and as high as 60 mg (Hallak et al., 1993) in some patients (inter-patient variability). Within the same patient (intra-patient variability), clinical (e.g., age, body weight, comorbidities, and physical activity levels) and environmental (e.g., drug-drug and drug-food interactions) factors can greatly influence the patient’s daily dose requirements (Turner and Pirmohamed, 2014). These dosing challenges usually result in a high frequency of adverse effects. Unsurprisingly, warfarin was the most commonly implicated drug in emergency hospitalizations for adverse drug events in older US adults between 2007 and 2009, having led to 33% of an estimated 99,628 annual hospitalizations (Budnitz et al., 2011). The requirement for more frequent monitoring also increases patient burden, which could impact quality of life and lead to the treatment discontinuation of an otherwise highly efficacious drug (Finkelman et al., 2016). In order to improve the quality of warfarin anticoagulation, several studies have been conducted to determine the factors that influence warfarin dose requirements (association studies) as well as develop dose-optimization algorithms (clinical prediction studies). However, these studies have mostly been conducted in Asian and White populations, as we recently reported for warfarin dose-optimization algorithms (Asiimwe et al., 2021).

**FIGURE 1:**
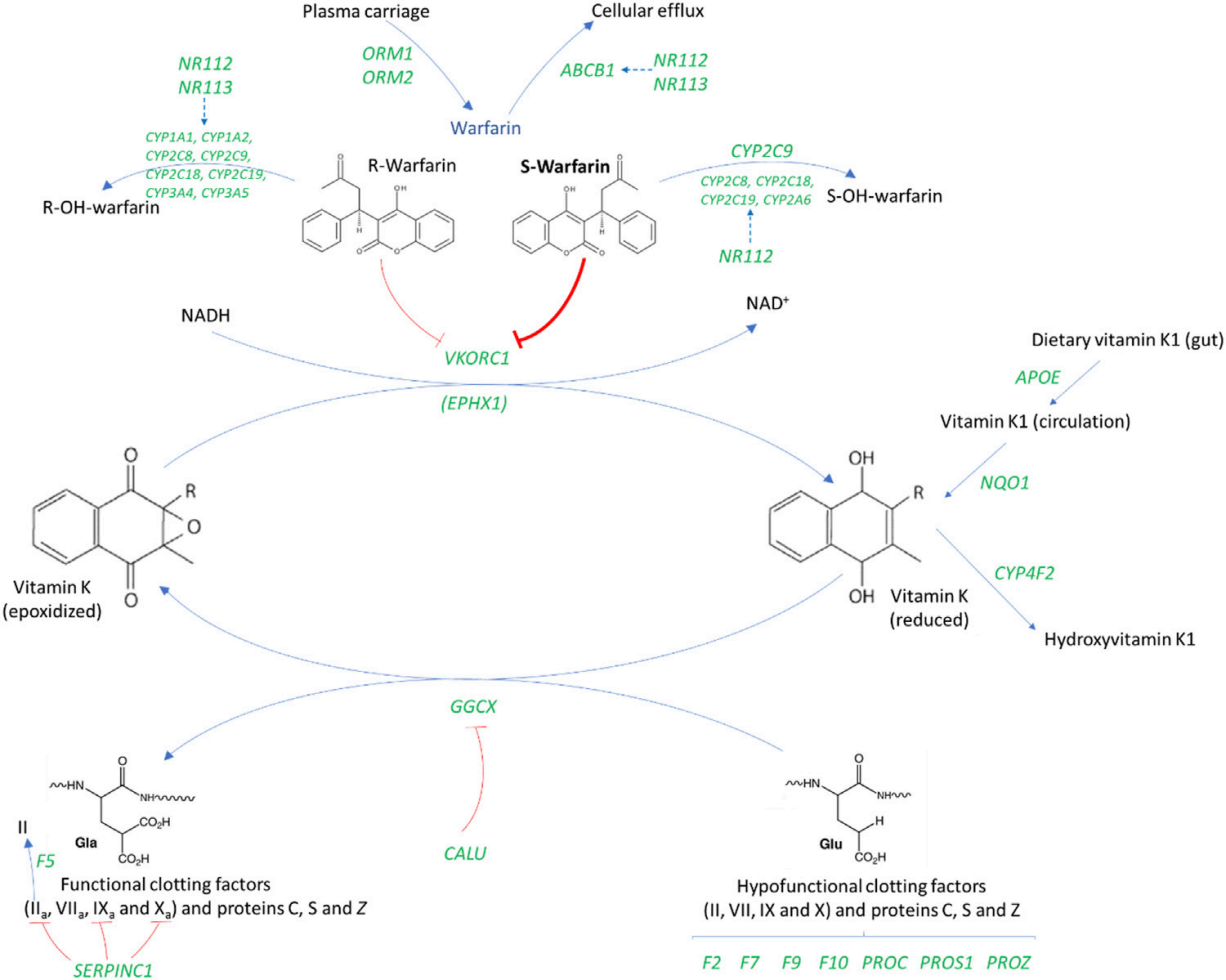
An overview of the genes involved in warfarin’s mechanism of action (Wadelius et al., 2007; Wadelius and Pirmohamed, 2007). *ABCB1*, ATP Binding Cassette Subfamily B Member one; *APOE*, Apolipoprotein E; *CALU*, Calumenin; *CYP*, Cytochrome P450; *EPHX1*, Epoxide hydrolase one; *GGCX*, Gamma-glutamyl carboxylase; *F2*, Coagulation factor II gene or prothrombin; *F5*, Coagulation factor V gene; *F7*, Coagulation factor VII gene; *F9*, Coagulation factor IX gene; *F10*, Coagulation factor X; *NQ O 1*, NAD (P) H dehydrogenase, quinone one; *NR1I2/3*, Nuclear Receptor Subfamily 1 Group I Member 2/3; *ORM*, Orosomucoid; *PROC*, Protein C; *PROS1*, Protein S; *PROZ*, Protein Z; *SERPINC1*, Serpin Family C Member one; *VKORC1*, Vitamin K epoxide reductase complex subunit 1. Gene names are italicized and shown in green.

Pharmacogenetics, the study of the genetic basis of variability in drug response (Nebert, 1999), was first coined in 1959 by the German Pharmacologist Friedrich Vogel (Vogel, 1959). Pharmacogenomics, a related term, was introduced in 1997 (Marshall, 1997) and although it is currently used interchangeably with pharmacogenetics (which usually focuses on a specific gene/set of genes), it is a broader-based term encompassing all genomic genes that may influence drug response (Pirmohamed, 2001; Dickmann and Ware, 2016). Despite these subtle differences in definition, both terms are part of personalized medicine and aim to improve the use of drugs from the present ‘trial-and-error’ approach to a more precise one where drugs are given to patients who stand to benefit the most and experience the least toxicity (Pirmohamed, 2011). Warfarin has been regarded as the poster child for pharmacogenomics (Pirmohamed, 2014) and is to date, the most studied drug in terms of genotype-phenotype relationships (Pirmohamed et al., 2015). It is an ideal candidate for the application of pharmacogenomics because it is a widely-used drug in addition to having dosing challenges (specifically wide interpatient variability) which could be minimized by a deeper understanding of the genes involved in its mechanism of action (Tan et al., 2010). More than 30 genes may be involved in warfarin’s pharmacokinetic and pharmacodynamic pathways including warfarin’s transport and biotransformation, and distribution of vitamin K, its hepatic uptake and metabolic pathways (Figure 1, Supplementary Table S1). The most important two genes in warfarin’s pharmacokinetics and pharmacodynamics are respectively *CYP2C9* (cytochrome P450 family two subfamily C polypeptide 9) and *VKORC1* (vitamin K epoxide reductase complex subunit 1) (Wadelius and Pirmohamed, 2007). As shown in Figure 1, *CYP2C9* encodes an enzyme that metabolizes the more potent enantiomer of warfarin (S-warfarin) while *VKORC1* encodes warfarin’s molecular target. Warfarin inhibits the vitamin K epoxide reductase protein resulting in decreased regeneration of reduced vitamin K that is required as a cofactor during the posttranslational carboxylation and activation of clotting factors II, VII, IX and X. Studies conducted in Whites have shown that *CYP2C9* and *VKORC1*, together with age, height, weight and interacting drugs account for approximately 50% of the variability in warfarin daily dose requirements (Wadelius and Pirmohamed, 2007; Lee and Klein, 2013). Due to differences in minor allele frequencies (MAFs) of key single nucleotide polymorphisms found in these genes (MAFs for the 1,000 genomes populations (Genomes Project et al., 2015) shown in Supplementary Table S1), the most important warfarin pharmacogenomic variants will differ from population to population (Cavallari and Perera, 2012). Failure to account for these differences can have dire consequences as demonstrated by the Clarification of Optimal Anticoagulation through Genetics (COAG) trial (Kimmel et al., 2013). Specifically, using a pharmacogenomic dosing algorithm developed from a majority White population led to poor performance in African Americans [compared to the clinically guided group (43.5%), African Americans in the genotype-guided group had a significantly lower mean percentage of time in the therapeutic range (35.2%, *p* = 0.01)] (Kimmel et al., 2013).

Because ethnicity is a social construct, its definitions are numerous, ambiguous and constantly evolving (Hamer et al., 2020; Brett and Goodman, 2021). For example, ethnicity may be defined in terms of race, culture, geographic location, country of origin, shared history/experiences, social class, population subgroups, biological references (genes, descent or physical appearance), among others (Hamer et al., 2020). The United States Office of Management and Budget specifies a minimum of five categories of self-reported race (American Indian or Alaska Native, Asian, Black or African American, Native Hawaiian or Other Pacific Islander, and White) and two ethnicity categories (Hispanic/Latino and Not Hispanic/Latino) (Office of Management and Budget, 1997). To avoid using racial/ethnic categories which incorporate complex sociocultural interactions and may be poorly defined, many geneticists rely on statistical genetic methods which enables the categorization of participants based on ‘genetic ancestry’ with individuals of a similar genetic makeup being classified together (Fujimura and Rajagopalan, 2011). Despite correlation, genetic ancestry captures different information from race/ethnicity and, as a result, genetic categories may not correspond to race/ethnic classifications (Fujimura and Rajagopalan, 2011; Borrell et al., 2021). Indeed, ethnic/racial categories should not be interpreted to represent biological or genetic differences (Office of Management and Budget, 1997).

Despite a lack of agreement in definitions, there is general agreement that the available pharmacogenomics evidence should reflect diversity in order not to exacerbate health inequalities (Borrell et al., 2021; Davis and Limdi, 2021; Oni-Orisan et al., 2021). In the field of warfarin pharmacogenomics, this is especially important as underrepresented groups also tend to be the most economically deprived (Williams et al., 2016), which means they are likely to be more reliant on warfarin for anticoagulation as it is currently the cheapest oral anticoagulant. This article therefore discusses warfarin pharmacogenomics, with a focus on whether individual races/ethnicities are represented in the existing body of evidence. To be consistent with one of the largest warfarin-related studies to date (International Warfarin Pharmacogenetics Consortium et al., 2009) and our recent systematic review (Asiimwe et al., 2021), the four major race/ethnic/population categories, which include the three major continental racial categories (Zhang and Finkelstein, 2019) are White (including Hispanic/Latino), Asian, Black, and Mixed/Other. Where relevant, countries of recruitment are used to explain population substructure.

## 2 Warfarin Pharmacogenomic Studies

**FIGURE 2:**
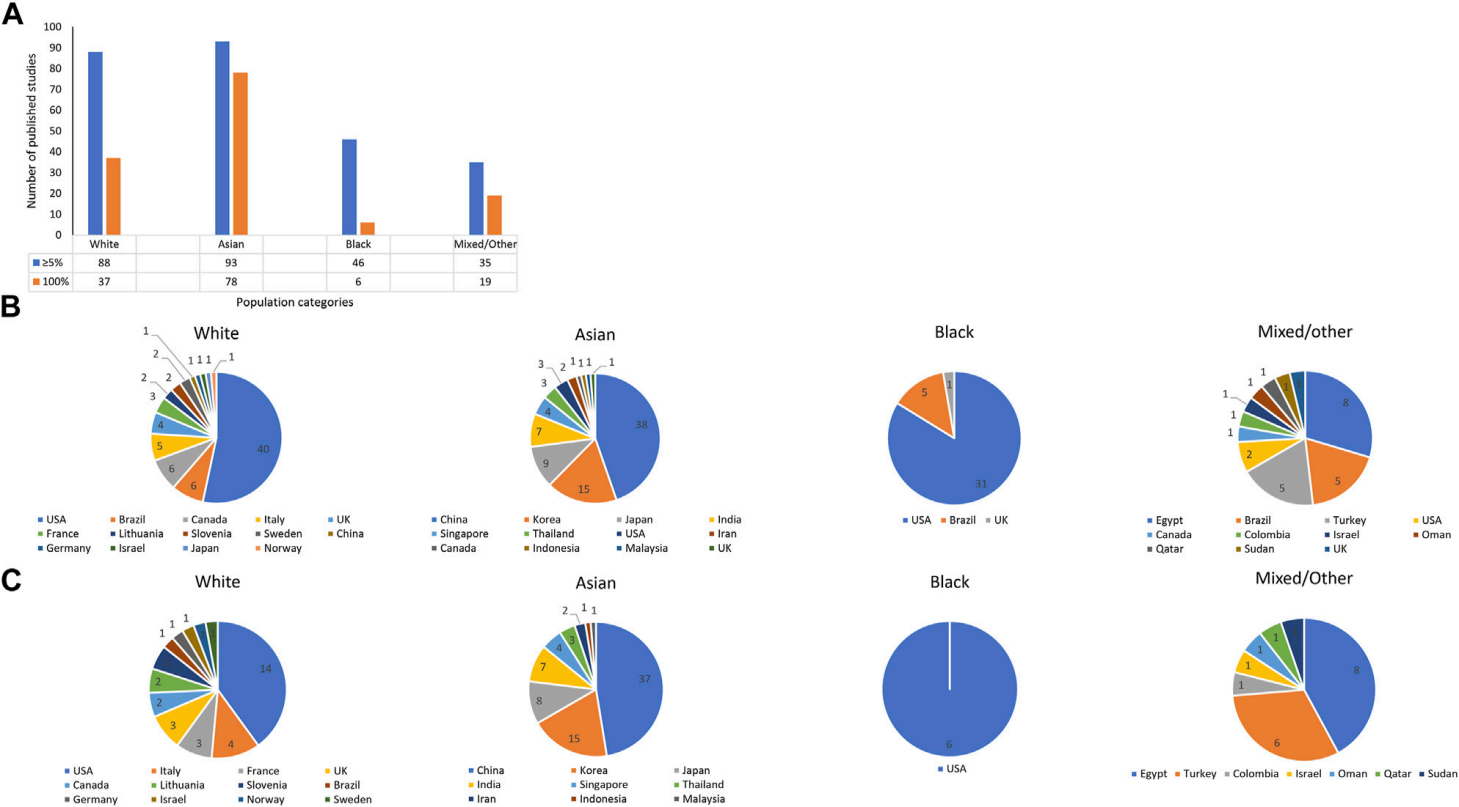
Number of pharmacogenomic studies that developed dosing algorithms as of 20 May 2020. **A** shows the number of pharmacogenomic studies that included at least 5% or 100% of a population category. On the other hand, **B, C** show the country-breakdown per population category with **B** corresponding to the 5% cut-off and **C**, the 100% cut-off. As recruitment was not stratified by location/country, studies that recruited from multiple countries are excluded in **B, C**.

Warfarin pharmacogenomic studies (both association and clinical prediction studies) are currently estimated to be in the thousands (Davis and Limdi, 2021). Due to the tremendous number of publications, our group recently conducted a more focused systematic review that included only clinical prediction studies/studies developing warfarin dosing algorithms (Asiimwe et al., 2021), and this provides the starting ground to understand ethnic diversity in the field of warfarin pharmacogenomics. As of 20 May 2020, 191 studies reporting the development of warfarin pharmacogenomic dosing algorithms had been published (Figure 2, Panel A). When an assumption was made that a study was applicable to a population category if it recruited at least 5% of participants from that category, then 88 (46%), 93 (49%), 46 (24%), and 35 (18%) algorithms were applicable to White, Asian, Black, and Mixed/Other populations, respectively. Race-stratified analyses are crucial to identifying any differential effects of key genetic (and non-genetic) factors among the different races/ethnicities–however, the different races/ethnicities should be well-represented in a trial’s total sample size (Davis and Limdi, 2021). As a 5% cut-off might not facilitate subgroup analysis when the overall sample size is low (Allmark, 2004), Figure 2 also includes a count of studies that recruited 100% of a particular population.

### 2.1 Whites

Close to half (46%) of the pharmacogenomic studies in Figure 2 recruited at least 5% of populations categorized as White, with most studies being conducted in the United States (*n* = 40, with five from Puerto Rico), Brazil (*n* = 6), Canada (*n* = 6), and Italy (*n* = 5). It is important to note that this category previously included non-Black Hispanics (Asiimwe et al., 2021), and although Whites are relatively homogeneous, they also have population substructure. For example, using principal component analysis, Northern Europeans (e.g., the Finnish) can be differentiated from Southern Europeans (e.g., Italians), Western Europeans (e.g., the British) and Caucasians outside of Europe (e.g., Israeli Caucasians) (Elhaik et al., 2013).

### 2.2 Hispanics/Latinos

Hispanics/Latinos, if present and not classified as Black were included under the White category in our previous review (Asiimwe et al., 2021). As Hispanics/Latinos are also considered an underrepresented ethnicity (Cavallari and Perera, 2012; Kaye et al., 2017), they require specific mention. Studies from Brazil and Puerto Rico (United States) have previously been classified as Latino studies (Kaye et al., 2017), and if we take a similar approach, then 12 (6%) of the warfarin pharmacogenomic studies included at least 5% Hispanics/Latinos, with six studies being conducted in Brazil, five studies in Puerto Rico and one study in Illinois (United States) (Asiimwe et al., 2021). Hispanics/Latinos may be considered admixed populations with a previous study that genotyped 93 ancestry informative markers in 642 Puerto Rican individuals estimating the average proportion of European, African and Native American ancestries as 63.7, 21.2 and 15.2%, respectively, (Via et al., 2011).

### 2.3 Asians

Almost half (49%) of the 191 pharmacogenomic studies recruited at least 5% Asians, with most studies being from China (*n* = 38), South Korea (*n* = 15), Japan (*n* = 9), and India (*n* = 7). As these numbers show, about three quarters of the studies are conducted in just four countries. Since Asian populations also have substructure (more details under the Genetic variants influencing warfarin response section), some Asian populations are currently underrepresented in the existing pharmacogenomics body of evidence.

### 2.4 Blacks

Studies that recruited at least 5% patients who identified themselves as Black were less than a quarter (24%) of the pharmacogenomic studies, being mostly conducted in the United States (*n* = 31) and Brazil (*n* = 5). Since African-Americans and African-Brazilians may not be representative of all Blacks (more details under the Genetic variants influencing warfarin response section), many Blacks are currently unrepresented in the current pharmacogenomic evidence.

### 2.5 Mixed/Other

The mixed/other category had the least amount of studies with only 18% of the pharmacogenomic studies recruiting this population category.

## 3 Genetic Variants Influencing Warfarin Response

Genome-wide association studies have confirmed that the most important warfarin pharmacogenomic variants are found in two genes, *CYP2C9* and *VKORC1* (Cooper et al., 2008; Takeuchi et al., 2009; Cha et al., 2010; Perera et al., 2013; Parra et al., 2015; El Rouby et al., 2021). It is also known that the most important variants will differ between populations, which has been mainly attributed to differences in minor allele frequencies (MAFs) of these variants (Cavallari and Perera, 2012; Johnson et al., 2017). With reference to summary information derived from our previous review (Asiimwe et al., 2021), this section looks at the variants most included in warfarin dosing pharmacogenomic algorithms, explores to what extent they influence warfarin response in the different populations, and points out key differences in MAFs between specific populations.

### 3.1 Whites

**FIGURE 3:**
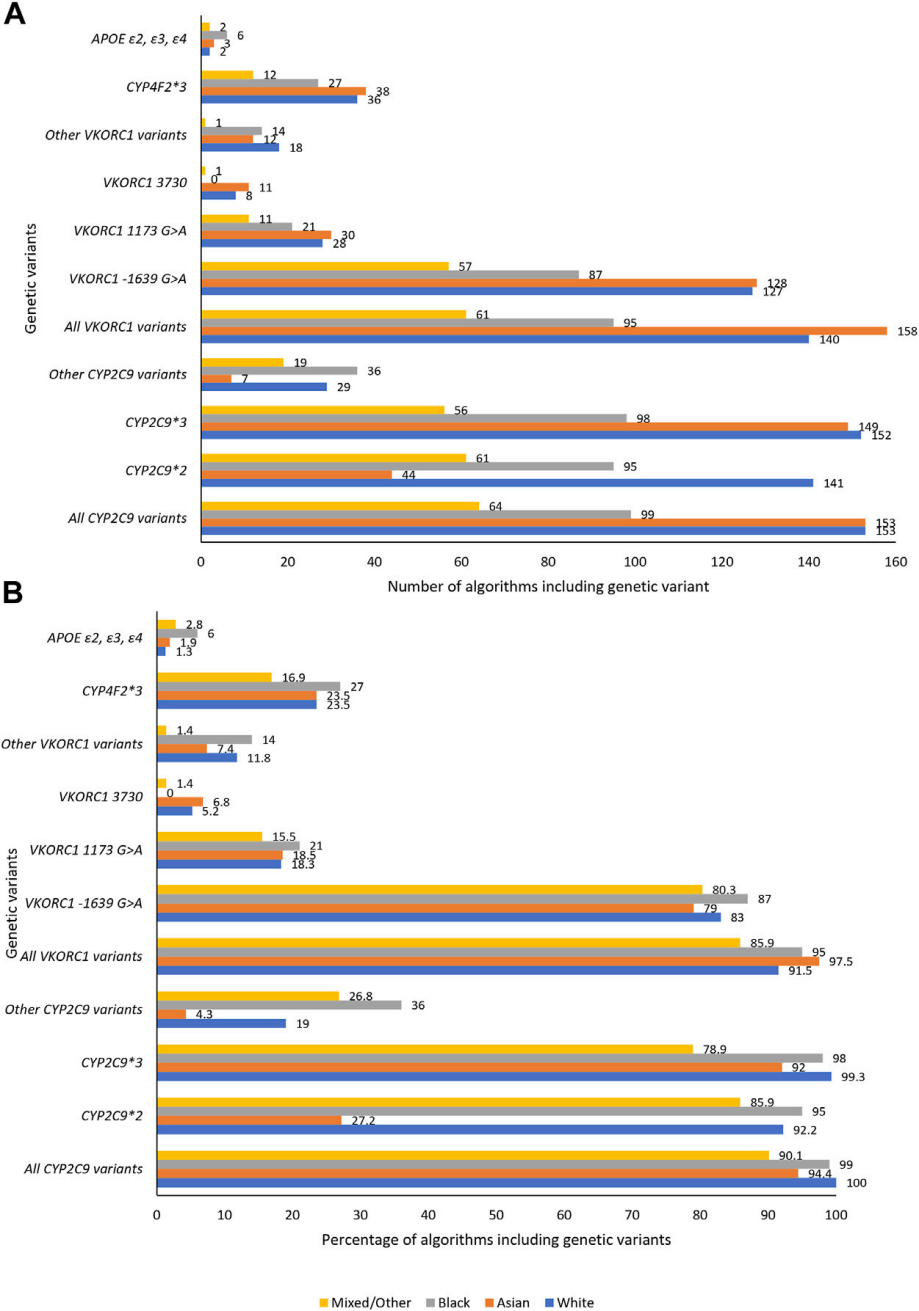
Genetic variables included in the pharmacogenomic algorithms. **A, B**, respectively, show the number and percentage of pharmacogenomic algorithms including a specific genetic variant. *APOE*, Apolipoprotein E; *CYP*, Cytochrome P450; *VKORC1*, Vitamin K epoxide reductase complex subunit 1.

One hundred fifty-three pharmacogenomic algorithms included at least 5% White participants—all included at least one *CYP2C9* variant while 92% algorithms included at least one *VKORC1* variant (Figure 3). CYP2C9*2 (included in 92% algorithms) and CYP2C9*3 (included in 99% algorithms) were the most included *CYP2C9* variants with other *CYP2C9* variants being included in only 19% of the algorithms. This low number (19%) is however not concerning as other *CYP2C9* variants (e.g., CYP2C9*5, CYP2C9*6, CYP2C9*8 and CYP2C9*11) are almost absent in populations of non-African, including European Ancestry [1000s genomes population MAF ∼0.00 (Genomes Project et al., 2015)]. Indeed, the most important *CYP2C9* genetic variants (CYP2C9*2 [MAF 0.12; reduces enzyme activity to about 12% of the wild-type and daily dose by up to 0.8 mg in Whites] and CYP2C9*3 [MAF 0.07; reduces enzyme activity to <5% of the wild-type and daily dose by up to 2.3 mg] (Rettie et al., 1994; Haining et al., 1996; Jorgensen et al., 2012; Genomes Project et al., 2015) are accounted for in almost all algorithms applicable to White populations. Similarly, the most important *VKORC1* genetic variant (*VKORC1 -1639G > A*) which reduces daily dose by up to 3 mg/day is accounted for either by being included (83% of algorithms) or by inclusion of another variant (*VKORC1 1173C > T*, 18% of algorithms) which is in near perfect linkage disequilibrium (both have MAF of 0.39 in populations of European Ancestry) (Jorgensen et al., 2012; Genomes Project et al., 2015). The 3′-untranslated region variant, *VKORC1 3730G > A* [MAF 0.37 in Europeans (Genomes Project et al., 2015)], which increases warfarin dose requirements through microRNA regulation (Perez-Andreu et al., 2013) was included in 5% of the algorithms while the CYP4F2*3 variant [found in the cytochrome P450 family four subfamily F member two gene that encodes a vitamin K oxidase (Caldwell et al., 2008)] was included in 24% of the algorithms. The lower inclusion of the CYP4F2*3 variant is consistent with previous reports that it influences only 1–2% in the variability in warfarin’s dose requirements (Takeuchi et al., 2009).

Table 1 shows the proportion of total interpatient variability in warfarin dose requirements (*R* ^*2*^) explained by genetic and non-genetic factors included in the warfarin pharmacogenomic dosing algorithms that reported this fit accuracy measure. For example, in 115 pharmacogenomic algorithms that included at least 5% of the White populations, the median variability in warfarin dose explained by both genetic and non-genetic factors included in these algorithms was 52% (range 20–82%), a figure which is consistent with earlier reviews (Wadelius and Pirmohamed, 2007; Lee and Klein, 2013). The respective contributions of the genes *CYP2C9* (40 algorithms) and *VKORC1* (44 algorithms) were median *R* ^*2*^ of 9% (range <1–50%) and 25% (range 1–52%). *CYP2C9*’s contribution (9%) may seem lower than previous estimates such as 12% (Baker and Johnson, 2016) and 15% (Pirmohamed et al., 2015) but this is explained by the fact that these figures also include warfarin variabilities of other populations. When only the algorithms that included only White patients are considered, the median variability attributed to the two genes *CYP2C9* (15 algorithms) and *VKORC1* (16 algorithms) becomes 12% (range <1–50%) and 27% (range 3–35%), estimates that are consistent with previous reports (Pirmohamed et al., 2015; Baker and Johnson, 2016).

**TABLE 1:** Proportion of variability explained by *CYP2C9* and *VKORC1* genes (Asiimwe et al., 2021).

	Population Categories							
	White		Asian		Black		Mixed/Other	
	N	% *R* ^2^, Median (Range)	N	% *R* ^2^, Median (Range)	N	% *R* ^2^, Median (Range)	N	% *R* ^2^, Median (Range)
≥5% of the population included								
All genetic and non-genetic factors	115	52 (20–82)	126	44 (11–96)	72	46 (22–82)	31	44 (8–77)
*CYP2C9* variants	40	9 (<1–50)	48	7 (<1–42)	21	7 (2–17)	23	8 (2–24)
*VKORC1* variants	44	25 (1–52)	54	27 (3–59)	24	23 (1–52)	27	20 (5–45)
100% of the population included								
All genetic and non-genetic factors	61	51 (20–70)	113	44 (11–96)	29	34 (22–66)	18	38 (8–62)
*CYP2C9* variants	15	12 (<1–50)	41	7 (<1–42)	1	9	13	8 (4–24)
*VKORC1* variants	16	27 (3–35)	47	27 (8–59)	3	9 (7–10)	17	20 (6–45)

CYP2C9, cytochrome P450 family 2 subfamily C polypeptide 9; N, number of algorithms; R2, R-squared (the coefficient of variation); VKORC1, vitamin K epoxide reductase complex subunit 1.

It is important to point out that MAFs are population averages that may differ within the same population depending on which participants are included in a particular study. For example, compared to the average European (MAF 0.12), the prevalence of the CYP2C9*3 has been reported to be statistically different (*p* < 0.05) in the Dutch (MAF 0.20), Polish (MAF 0.01) and Spanish (MAF 0.16) cohorts (Garcia-Martin et al., 2001; Mizzi et al., 2016). In another study that compared 845 healthy volunteers from four different Russian ethnic groups (238 Chuvash, 206 Marians, 157 Kabardians and 244 Ossetians) with another Russian population that comprised 400 atrial fibrillation patients, significant differences were identified in the CYP2C9*2 (ranging from 0.06 to 0.12), CYP2C9*3 (ranging from 0.07 to 0.14) and *VKORC1 -1639G > A* (ranging from 0.38 to 0.50) MAFs (Zotova et al., 2013; Mirzaev et al., 2020). Moreover, extrapolating a MAF from one population to another may not be straightforward as different variants may follow different geographical distribution patterns. For instance, Balanovskaya and others used a cartographic approach (2,197 participants representing 50 different populations/137 ethnic and subethnic groups) to visualize the geographic distribution of pharmacogenetic markers across Russia and its neighbouring countries and reported that: CYP2C9*2 followed a focal variation pattern [the CYP2C9*2 allele was specific to certain ethnicities (MAF as high as 0.23) and absent in others]; CYP2C9*3 followed a uniform pattern [MAF varying within a narrow range (0.03 to 0.10) in most ethnic groups]; and *VKORC1 -1639G > A* followed a clinal pattern [a gradient decrease in MAF (from >0.95 to ∼0.30) along the East-West axis] (Balanovskaya et al., 2020).

### 3.2 Hispanics/Latinos

Eleven algorithms from eight studies conducted in Brazil/Puerto Rico reported partial *R* ^*2*^ for *CYP2C9* or *VKORC1* (Asiimwe et al., 2021), with the median partial *R* ^*2*^ for *CYP2C9* and *VKORC1* being 7% (range 2–17%) and 23% (range 2–31%), respectively. The lower contribution of *CYP2C9* for Hispanics/Latinos (almost half that of Europeans) implies these populations may benefit less from CYP2C9*2 and CYP2C9*3 variants (that were included in all these algorithms) and may benefit more from the incorporation of additional variants. These additional variants (such as CYP2C9*5, CYP2C9*6, CYP2C9*8, and CYP2C9*11) have mainly been discovered from Blacks/Africans, meaning that their inclusion or exclusion is likely to depend on the proportion of African ancestry that is detected within a particular Hispanic/Latino cohort.

Consistent with being admixed populations, MAFs for Hispanics/Latinos are usually lower than those for Whites (decrease progressively with increasing individual proportion of African ancestry), with one large cohort of >1,000 Brazilians reporting MAFs of 0.11, 0.05, and 0.33 for CYP2C9*2, CYP2C9*3, and *VKORC1 -1639G > A*, respectively, (Rodrigues-Soares and Suarez-Kurtz, 2019). However, some MAFs may be higher in admixed populations for traits that have been under differential selection pressure among the ancestral populations or among deeply divergent populations (Lin et al., 2021).

### 3.3 Asians

One hundred sixty-two pharmacogenomic algorithms included at least 5% Asian participants, and for these 94% included at least one *CYP2C9* variant while 98% algorithms included at least one *VKORC1* variant (Figure 3). Consistent with being almost absent in some Asian populations [East Asian MAF ∼0.00, South Asian MAF 0.04 (Genomes Project et al., 2015)], CYP2C9*2 which reduces daily doses by up to 0.7 mg in Asians (Jorgensen et al., 2012) was included in only 27% of the 162 algorithms. Both CYP2C9*3 [East Asian MAF 0.03, South Asian MAF 0.11; reduces daily dose by up to 1.5 mg (Jorgensen et al., 2012; Genomes Project et al., 2015)] and *VKORC1 -1639G > A* [East Asian MAF ∼0.89, South Asian MAF 0.15; reduces daily dose by up to 1.5 mg (Jorgensen et al., 2012; Genomes Project et al., 2015)] were well-represented, being included in 92 and 79% of the algorithms respectively. *VKORC1 1173C > T* which is in near perfect linkage disequilibrium with *VKORC1 -1639G > A* was preferred in 19% of the algorithms. As for Whites, CYP4F2*3 [reported to influence only 1–2% in the variability in warfarin’s dose requirements in Japanese (Cha et al., 2010)] was included in 24% of the algorithms.

One hundred thirteen pharmacogenomic algorithms included only Asian populations (Table 1) with *CYP2C9* (41 algorithms) and *VKORC1* (47 algorithms) having median *R* ^*2*^ of 7% (range <1–42%) and 27% (range 8–59%), respectively. Compared to South Asians (CYP2C9*2 MAF 0.04; CYP2C9*3 MAF 0.11), East Asians (CYP2C9*2 MAF∼0.00; CYP2C9*3 MAF 0.03) have lower MAFs for these two *CYP2C9* variants. Indeed, when 32 algorithms from East Asia (18 from China, 12 from South Korea and two from Japan) were analysed, the median partial *R* ^2^ for *CYP2C9* was only 6% (range 1–33%). On the other hand, and despite only being two in number, South Asian algorithms (all from India) reported a median partial *R* ^2^ for *CYP2C9* of 27% (range 12–42%), which corresponds to a higher importance of these variants in the South Asians. Although the *VKORC1 -1639G > A* MAF for East Asians (MAF 0.89) is much higher than that of South Asians (MAF 0.15), the *R* ^*2*^ attributed to *VKORC1* is similar between the East and South Asians (East Asian median *R* ^*2*^ = 27%, range 8–49%, 39 algorithms [22 from China, 12 from South Korea and five from Japan]; South Asian median *R* ^*2*^ = 28%, range 23–32%, two algorithms from India). This is explainable by the fact that two alleles with MAFs of *p* and *q* (1-*p*) will have a similar variability if their effect sizes are the same. Therefore, the East Asian MAF of 0.89 will be equivalent to 1—0.89 = 0.11, which is comparable with the South Asian MAF of 0.15.

As shown above, the majority of existing pharmacogenomic algorithms are from East Asia (China, South Korea and Japan), which means Southern, South Eastern, Western, and other Asians may be currently underrepresented in the current pharmacogenomic knowledge base. For East Asia, most studies are from China, with most Chinese being recruited being Han Chinese, which is the largest subethnic group in China. However, other subethnic groups, such as Tu, Tujia and Xibo which for instance have a CYP2C9*3 MAF greater than 0.10, exist in China and these are currently underrepresented (Lam and Cheung, 2012). Additionally, with increasing urbanization and globalization, admixed populations including those arising between the different subethnicities and which are not as well-defined as homogeneous ethnic groups are on the increase (Lam and Cheung, 2012). On the other hand, most South Asian studies are Indian. India itself is genetically diverse–it is an intermixture of four main ancestral populations, the ancestral North Indians, ancestral South Indians, ancestral Tibeto-Burmans, and ancestral Austro-Asiatics (Basu et al., 2016; Sengupta et al., 2016). In one study, the CYP2C9*2 MAF was estimated to be 0.09 in 803 North Indians (highest in 375 individuals from Lucknow, Utter Pradesh, MAF = 0.14) and 0.04 in 481 South Indians (lowest in 120 individuals in Kerala, MAF = 0.02) (Umamaheswaran et al., 2014). In another study that re-analyzed genotype microarray datasets comprising 2,680 individuals across 24 ethnically diverse Indian subpopulations, *VKORC1 -1639G > A* was highly variable across these populations with a MAF <0.07 in an out-group subpopulation to one >0.70 in Tibeto–Burmans (Giri et al., 2014).

### 3.4 Blacks

Ninety-nine percent and 95% of the 100 pharmacogenomic algorithms that included at least 5% Black participants included at least one *CYP2C9* or *VKORC1* variant respectively (Figure 3). CYP2C9*2 [MAF 0.01 (Genomes Project et al., 2015)] and CYP2C9*3 [MAF ∼0.00 (Genomes Project et al., 2015)] variants were, respectively, included in 95 and 98% of the algorithms, despite the low population frequencies. In terms of personalized medicine, their inclusion in these algorithms is important since these variants are still important at an individual level. For example, in a previous systematic review, we showed that individuals of Black-African ancestry who are heterozygous for either CYP2C9*2 or CYP2C9*3 variants will have weekly dose reduced by either 6.8 or 12.5 mg, respectively, (Asiimwe et al., 2020), effects which are similar to those observed in White patients [respective weekly dose reductions of 3.9 and 12.5 mg (Jorgensen et al., 2012)]. To avoid sub-optimal anticoagulation, it is crucial that all important genetic variants, such as CYP2C9*2 and CYP2C9*3 discussed above, are accounted for in algorithms (Pratt et al., 2019).

In Black-Africans, it is now recognized that other *CYP2C9* variants, such as CYP2C9*5, CYP2C9*6, CYP2C9*8, and CYP2C9*11, are important (Cavallari and Perera, 2012; Asiimwe et al., 2020). Unfortunately, only 36% of the algorithms included at least one other *CYP2C9* variant that was not CYP2C9*2 or CYP2C9*3, which means most of the studies did not account for these important variants. It is recognized that some studies may not include these variants due to reasons related to power, however, they can be combined into a single group comprising *CYP2C9* star variant alleles (Perera et al., 2011), which increases power. A SNP found in the *CYP2C* gene cluster region (rs12777823, African MAF 0.25) has been associated with dose reduction of up to 12.7 mg/week in patients of Black-African ancestry (Asiimwe et al., 2020); however it was only included in 19 algorithms from six studies. Importantly, dose reduction with this SNP is only observed in Africans (Perera et al., 2013) despite being common in other populations (Genomes Project et al., 2015). The two *VKORC1* variants which are in high linkage disequilibrium and reduce weekly dose by up to 18.1 mg (*VKORC1 -1639G > A*) and 21.6 mg (*VKORC1 1173C > T*) (Asiimwe et al., 2020) were well-represented, respectively, appearing in 87 and 21% of algorithms (the total is more than 100% since for 13 algorithms, these SNPs, being in perfect linkage disequilibrium, were interchangeably used or both mentioned). Despite increasing weekly warfarin dose by up to 6.9 mg (Asiimwe et al., 2020) and having a MAF of 0.45 in Black-Africans (Genomes Project et al., 2015), *VKORC1 3730G > A* was not included in any of the pharmacogenomic dosing algorithms applicable to Black populations. Lastly, CYP4F2*3 appeared in 27% of the algorithms despite not being associated with warfarin dose requirements in our review (Asiimwe et al., 2020). Given that it only explains an additional 1–2% of observed warfarin dose variability in Caucasians (MAF 0.29) (Takeuchi et al., 2009) and East Asians (MAF 0.21) (Cha et al., 2010), its effects are likely to be even lower in Africans who have a MAF of only 0.08.

Despite genetic and non-genetic variables explaining more than 50% of warfarin dose variability in White patients, these variables could only explain a median of 34% (range 22–66%) of warfarin dose variability in the algorithms (*n* = 29) that included only Black populations (Table 1). Only one and three algorithms, all from the United States, respectively, reported the partial *R* ^*2*^ for *CYP2C9* (*R* ^*2*^ of 9%) and *VKORC1* (median *R* ^*2*^ of 9%, range 7–10%). Despite the low numbers, the percentage contribution of these variants is quite low, which can be explained by the low MAFs of the *CYP2C9* variants. For example, the one study that reported the partial *R* ^*2*^ for *CYP2C9* (Perera et al., 2011) included the *CYP2C9* star variants CYP2C9*2 (study MAF 0.02), CYP2C9*3 (study MAF 0.01), CYP2C9*5 (study MAF 0.01), CYP2C9*8 (study MAF 0.06), and CYP2C9*11 (study MAF 0.04) or a combined MAF of 0.14 or less (some patients can have two star alleles). The univariable and partial *R* ^*2*^ attributed to these five star variants were 5.6 and 7.7%, respectively, [in Whites, CYP2C9*2 (MAF 0.12) and CYP2C9*3 (MAF 0.07) have an *R* ^*2*^ of about 12%]. If only the CYP2C9*2 and CYP2C9*3 variants had been analysed in the Perera et al. study, then the contribution of *CYP2C9* to warfarin variability could have been even smaller, which further demonstrates why important variants should not be excluded. In this same study *VKORC1 1173* (study MAF 0.12) had a partial *R* ^*2*^ of 7.4% (univariable *R* ^*2*^ of 9.9%), which is much smaller than the approximately 27% attributed to *VKORC1 1173* (which is in LD with *VKORC1 -1639G > A*) in Whites (MAF 0.39).

As we previously reported (Asiimwe et al., 2020), most existing evidence applicable to Blacks comes from African Americans and Brazilians who are of West African ancestry and who have admixed with European and Amerindian populations (Suarez-Kurtz and Botton, 2013), and so may not be generalizable to other Black African populations, such as those in sub-Saharan Africa. In Brazil, a substantial proportion of Amerindian (mean 7%, 95% CI 6%–9%) and European (mean 42%, 95% CI 36%–48%) ancestries were reported in 109 Brazilians who identified as Black (mean African ancestry 51%, 95% CI 45%–57%) (Suarez-Kurtz et al., 2007) while in the United States, some individuals who self-identify as African American have almost no African ancestry (Bryc et al., 2010). It is no surprise that the *VKORC1 -1639G > A* MAF of 0.05 in the 1,000 genomes African super-population is 2–5 times higher in African Americans (0.15) compared to sub-Saharan African sub-populations (Yoruba in Nigeria, 0.03; Mende in Sierra Leone, 0.05; Luhya in Kenya, 0.04; Gambian in Gambia, 0.07; and, Esan in Nigeria, 0.03) (Genomes Project et al., 2015). Additionally, sub-Saharan Africans are extremely diverse with genetic differences between sub-Saharan African ancestries sometimes exceeding those between pairs of non-African ancestries (Tishkoff et al., 2009; Shriner et al., 2014). Countries in sub-Saharan Africa such as South Africa also have admixed/mixed-ancestry populations (Muyambo et al., 2021) that may have unique genetic structures, despite self-identifying as Black.

### 3.5 Mixed/Other

This group comprised participants who were classified as ‘mixed’, ‘other’ or ‘unknown’ in the primary studies or studies, which could not be allocated to any of the three major population categories (‘White’, ‘Asian’, and ‘Black’). Of the 71 algorithms that included at least 5% Mixed/Other participants, 90, 86 and 17% algorithms included at least one *CYP2C9* variant, at least one *VKORC1* variant and CYP4F2*3, respectively, (Figure 3). Eighteen pharmacogenomic algorithms included only populations that were categorized as mixed/other (Table 1) with *CYP2C9* (13 algorithms) and *VKORC1* (17 algorithms) having median *R* ^*2*^ of 8% (range 4–24%) and 20% (range 6–45%), respectively. The countries from which these 18 pharmacogenomic algorithms were developed included Egypt (9 algorithms), Turkey (6 algorithms), and Colombia, Oman, and Sudan (1 algorithm each).

#### 3.5.1 Egypt

Egypt is found in the north of Africa but was not included with Blacks as one study that carried out genome-wide single analysis in six North African countries reported that these populations share more genomic ancestry with those in the Near East compared to those from sub-Saharan Africa (Henn et al., 2012). Although the Near East is considered part of West Asia (Marsden and Mostowlansky, 2019), Egypt could not be considered under Asians due to its geographical location, which resulted in it being included in this group. For six algorithms that reported the *CYP2C9* partial *R* ^2^, the median *R* ^2^ was 8% (range 5–16%) which is similar to that of the Blacks. This result is quite surprising since the MAFs of *CYP2C9* star variants may be more similar to Europeans than to Blacks. For example, in one study (Shahin et al., 2011), CYP2C9*2, CYP2C9*3, CYP2C9*4, CYP2C9*5, and CYP2C9*8 variants had MAFs of 0.12, 0.09, <0.01, 0.01, and <0.01, respectively, and yet the partial *R* ^2^ was only 5%, results which require further study. For eight algorithms that reported the *VKORC1* partial *R* ^2^, the median *R* ^2^ was 14% (range 7–32%) which is about half of the 27% for Whites. This result may again be surprising as the Shahin et al. study which reported a partial *R* ^2^ of 10% also reported a MAF of 0.46 for *VKORC1 -1639G > A* (Shahin et al., 2011), which is higher than 0.39 in Whites. Nevertheless, the important genetic determinants in Egyptian populations may be similar to other African populations. For example, a published abstract (a systematic review of pharmacogenetic studies in patients from any of the African countries) reported that based on mostly Egyptian studies (10 of 14 included studies), the genetic variants significantly associated with warfarin dose variability included *VKORC1 -1639G > A*, CYP2C9*2, CYP2C9*3, CYP2C9*5, CYP2C9*8, and CYP2C9*11 among others (Salem et al., 2018).

#### 3.5.2 Turkey

Turkish populations comprise different ethnic groups that harbour both European and Asian ancestry (Oner Ozgon et al., 2008; Ozer et al., 2010). The median *CYP2C9* partial *R* ^2^ from four Turkish studies was 16% (range 8–24%) while the median *VKORC1* partial *R* ^2^ from six Turkish studies was 22% (range 6–34%). The median *CYP2C9* partial *R* ^2^ of 16%, which is slightly higher than that for Whites (12%) may be unsurprising as MAFs for CYP2C9*3 have been reported to be higher than 0.07, for example two Turkish studies reported MAFs as 0.10 and 0.15 (Oner Ozgon et al., 2008; Ozer et al., 2010). The *VKORC1 -1639G > A* MAFs reported for the above two studies are respectively 0.50 and 0.40, which are higher or similar to the 1,000 genomes European MAF of 0.39. Surprisingly, the *VKORC1* partial *R* ^2^ of 22% is slightly lower than that for Whites (27%), which demonstrates why *R* ^2^ estimates should be taken as approximate.

#### 3.5.3 Colombia

Colombians, who like Puerto Ricans or Brazilians, could be classified as Hispanic, are admixed populations with about 49, 37, 10 and 3.4% being categorized as being of Mestizo (both European and Amerindian), European, African, and Amerindian ancestries, respectively, (De Castro and Restrepo, 2015). One study reported both the *CYP2C9* (4%) and *VKORC1* (26%) partial *R* ^*2*^ values, which were somewhat consistent with the reported MAFs (CYP2C9*2, CYP2C9*3, and *VKORC1 -1639G > A* MAFs were 0.07, 0.03, and 0.44, respectively) (Galvez et al., 2018).

#### 3.5.4 Oman

Omanis are a genetically admixed population comprising Caucasian, African and Asian ancestries (Pathare et al., 2012). In the Pathare et al. (2012) study that reported *CYP2C9* (17%) and *VKORC1* (45%) partial *R* ^*2*^ values, the CYP2C9*2, CYP2C9*3, CYP2C9*8, and *VKORC1 -1639G > A* MAFs were 0.08, 0.06, <0.01 and 0.38, respectively, which means the partial *R* ^*2*^ values are higher than those reported for Whites despite having similar MAFs. However, these estimates are from one study only.

#### 3.5.5 Sudan

Sudanese, who comprise Nilotic African tribes in the South and Arab-Africans in the North (Shrif et al., 2011) could be described as being of Black-African ancestry. However, due to their Arabic component, they were placed in this group as the Egyptians described above. The Shrif et al. study reported *CYP2C9* (*2, *5, *6, *11) and *VKORC1* (*1542G > C*, *3730G > A*, *rs7199949*) partial *R* ^*2*^ values of 5 and 27%, respectively. As Shrif and others report, the MAFs for CYP2C9*3 (0.00), CYP2C9*5 (0.01), CYP2C9*6 (0.02), and CYP2C9*11 (0.05) variants were comparable to those of Black Africans and African Americans, while the MAF for CYP2C9*2 (0.05) was between that of West/South Africans and that of North Africans/Caucasians. The MAF for *VKORC1 -1639G > A* (0.37) was similar to that for Whites, although this variant wasn’t included in the dosing algorithm.

## 4 Clinical Utility Studies

Before an algorithm can be recommended for use in clinical practice, it should be assessed for clinical utility, preferably using randomized control trials (RCTs) which are the current gold standard for evidence synthesis. Clinical utility (Grosse and Khoury, 2006), in this context, can be defined as an improvement in the quality of anticoagulation (usually based on the time spent in the therapeutic INR range) or a demonstration of better clinical endpoints (such as fewer haemorrhagic events). This section looks at which RCTs have been conducted in the various populations to see which algorithms are ready for implementation. As of 20 May 2020, 23 RCTs assessing the clinical utility of pharmacogenomic dosing algorithms had been published, which represented a total of 8,487 warfarin-treated patients (Asiimwe et al., 2021). To provide more up-to-date figures, we have included additional RCTs from a recent systematic review (Wang et al., 2022), which gives a total of 10,046 warfarin-treated patients who were randomized to either genotype-guided dosing or a control arm (Table 2). Table 2 focuses on whether the different populations are represented and the reader is referred to previous publications (Tse et al., 2018; Wang et al., 2022) for more details on the study results and/or quality.

**TABLE 2:** Randomized control trials^a^ assessing clinical utility, as of July 2021 (Asiimwe et al., 2021; Wang et al., 2022).

Clinical Utility study	Country	Sample Size					Key pharmacogenomic Algorithm
		Total Randomized	Whites (Hispanic)	Asian	Black	Mixed/Other	
Hillman et al. (2005)	United States	38	38	—	—	—	Hillman equation (Hillman et al., 2004)
Anderson et al. (2007)	United States	200	189	—	—	11	Carlquist equation (Carlquist et al., 2006)
Huang et al. (2009)	China	142	—	142	—	—	Huang equation (Huang et al., 2009)
Burmester et al. (2011)	United States	230	230 (not stated)	—	—	—	Caldwell algorithm (Caldwell et al., 2008)
Korneva et al. (2011)	Russia	61	61^b^	—	—	—	No information (abstract)
Borgman et al. (2012)	United States	26^c^	24	—	—	2	PerMIT tool (Linder et al., 2009)
Radhakrishnan et al. (2012)	United States	56	—	—	—	—	No information (abstract)
Wang et al. (2012)	China	106	—	106	—	—	Huang equation (Huang et al., 2009)
Jonas et al. (2013)	United States	109	79	—	30	—	WarfarinDosing.org (Gage et al., 2008)
Kimmel et al. (2013)	United States	1,015	740 (65)	—	275	—	WarfarinDosing.org (Gage et al., 2008)
Li et al. (2013)	China	220	—	220	—	—	No information (English abstract)
Pirmohamed et al. (2013)	United Kingdom and Sweden	455	447	2	5	1	Modified IWPC equation (International Warfarin Pharmacogenetics Consortium et al., 2009)
Pengo et al. (2015)	Italy	200	200	—	—	—	Zambon equation (Zambon et al., 2011)
Duan et al. (2016)	China	55	—	55	—	—	No information (abstract)
Jiang et al. (2016)	China	60	—	60	—	—	Jiang equation (Jiang et al., 2016)
Pavani et al. (2016)	India	207	—	207	—	—	Artificial neural network model (Pavani et al., 2016)
Gage et al. (2017)	United States	1,650	1,502 (42)	29	106	13	WarfarinDosing.org (Gage et al., 2008)
Jin et al. (2017)	China	238	—	238	—	—	WarfarinDosing.org (Gage et al., 2008)
Wen et al. (2017)	Taiwan (China)	318	—	318	—	—	Wen (Wen et al., 2008) and IWPC (International Warfarin Pharmacogenetics Consortium et al., 2009) equations
Jiang et al. (2018)	China	87	—	87	—	—	Lou equation (Jiang et al., 2018)
Makar-Ausperger et al. (2018)	Croatia	205	205^b^	—	—	—	WarfarinDosing.org (Gage et al., 2008)
Syn et al. (2018)	Singapore and Malaysia	322	—	322	—	—	Tham equation (Tham et al., 2006)
Xu et al. (2018)	China	201	—	201	—	—	Cen equation (Cen et al., 2010)
(Al-Metwali et al., 2019)^d^	United kingdom	26^e^	20	4	—	2	A set of differential equations (Hamberg et al., 2013)
Hao et al. (2019)	China	2,264	—	2,264	—	—	IWPC equation (International Warfarin Pharmacogenetics Consortium et al., 2009)
Guo et al. (2020)	China	660	—	660	—	—	Modified IWPC equation (International Warfarin Pharmacogenetics Consortium et al., 2009)
Lee et al. (2020)	South Korea	125	—	125	—	—	Lee equation (Lee et al., 2012)
Panchenko et al. (2020)	Russia	263	263	—	—	—	WarfarinDosing.org (Gage et al., 2008)
Zhu et al. (2020b)	China	507	—	507	—	—	Zambon equation (Zambon et al., 2011)
Total (n, %)		10,046	1,998, 39.8	1,547, 55.2	416, 4.1	29, 0.3	

a Some trials are reported as randomized, but published reports did not provide enough information to verify this.

b Ethnicity/race not stated but participants recruited from a majority White country.

c Represents the analyzed population (34 were randomized but details on ethnicity/race unavailable).

d Recruited children.

e Represents the analyzed population (29 were randomized but details on ethnicity/race unavailable).

IWPC, international warfarin pharmacogenetics consortium; PerMIT, personalized medicine interface tool; UK, United Kingdom; USA, United States of America.

### 4.1 Whites

More than a third (40%) of the participants randomized to RCTs evaluating the clinical utility of genotype guided dosing identified as White. The three largest trials that recruited this population were the Genetic Informatics Trial (GIFT) conducted in the United States (Gage et al., 2017), the Clarification of Optimal Anticoagulation through Genetics (COAG) trial also conducted in the United States (Kimmel et al., 2013) and the European Pharmacogenetics of Anticoagulant Therapy (EU-PACT) trial conducted in the United Kingdom and Sweden (Pirmohamed et al., 2013). Two of these trials (COAG and EU-PACT) were published at the same time in 2013 and many comparisons were made between them because of contradictory results. Whereas EU-PACT (*n* = 427 analysed patients) found a benefit with pharmacogenomic-guided dosing (improved percent time in therapeutic range (PTTR), shorter time to stable dose, and reduced number of episodes with an INR >4), COAG (*n* = 955) did not (no differences in PTTR, time to stable dose and reduction in number of episodes with INR >4 or <2). These differences were attributed to different algorithmic strategies (loading dose algorithm in EU-PACT versus maintenance dose algorithm in COAG, reflecting clinical practices in Europe and the United States), ethnic heterogeneity (almost homogeneous [99% Caucasian] population in EU-PACT versus an ethnically diverse cohort that included 27% African-Americans in COAG) and comparator arms (fixed dosing versus clinically-guided dosing) among other reasons (Pirmohamed et al., 2015). For all patients (*n* = 955), genotype-guided dosing changed the mean PTTR by −0.2 (95% CI −3.4 to 3.1, *p* = 0.91) and although it was still not statistically significant, it changed direction when Blacks were excluded from analysis [for non-Blacks (*n* = 700), the mean PTTR increased by 2.8 (95% CI −1.0 to 6.6, *p* = 0.15)]. The third trial (GIFT) provided more clarity on the benefit of genotype-guided dosing in patients ≥65 years who were starting warfarin for elective hip or knee arthroplasty. A comparison between the genotype-guided group (*n* = 803) and the clinically-guided group (*n* = 785) showed that genotyping significantly improved the PTTR over 4 weeks (mean difference 3.4, 95% CI 1.1 to 5.8, *p* = 0.004), with the effect being slightly more pronounced in non-Blacks (751 vs. 735 patients, mean difference 3.7, 95% CI 1.2 to 6.1, *p* = 0.003). Genotype-guided dosing also reduced the combined risk of major bleeding, INR ≥ 4, venous thromboembolism, or death (absolute difference 3.9%, 95% CI 0.7%–7.2%; relative rate 0.73, 95% CI 0.56 to 0.95, *p* = 0.02).

### 4.2 Hispanics/Latinos

Three studies (Burmester et al., 2011; Kimmel et al., 2013; Gage et al., 2017) reported recruiting Hispanics, although one (Burmester et al., 2011) did not report the number of ‘White Hispanics’ enrolled. From the two studies, it can be estimated that at least 107 Hispanics were represented, although this figure is very low (1% of the 10,046 patients).

### 4.3 Asians

With more than a half (55% of 10,046) of the participants, Asians are the most represented in the RCTs assessing the clinical utility of pharmacogenomic algorithms. However, this may be an underrepresentation if you consider that Asians make up about 60% of the world’s population (Lo et al., 2020). Additionally, this representation is mostly attributed to one recent large-sized trial (Hao et al., 2019) that randomized 2,264 patients or 41% of all Asian patients. In this trial that recruited Chinese patients with heart valve replacement, genotype-guided dosing reduced the number of days to reach therapeutic INR (genotype-guided dosing vs. clinically-guided dosing, 3.8 ± 2 vs. 4.4 ± 2, *p* < 0.001) although there was no reduction in major bleeding or thrombotic events. Two other recent relatively-large trials, respectively, randomized 660 and 507 patients and both reported that genotype-guided dosing was better than clinically-guided dosing in terms of the PTTR [respective mean differences 5.6 (95% CI 1.1 to 10.2, *p* = 0.01) and 17.4 (95% CI 11.8 to 22.9, *p* < 0.01)] (Zhu et al., 2020b; Guo et al., 2020). These trials together with other Chinese trials recruited 4,858 patients or 88% of all Asian participants meaning other Asian countries/non-Chinese populations are currently underrepresented. Other Asians were recruited from Singapore/Malaysia (*n* = 322, 6%), India (*n* = 207, 4%), South Korea (*n* = 125, 2%), and United Kingdom/United States (*n* = 35, <1%).

### 4.4 Blacks

With only 416 (4% of 10,046) patients, Blacks are underrepresented in terms of evidence of clinical utility. Four hundred eleven (99% of the Black) patients were recruited in the United States with the most relevant trials being the COAG (Kimmel et al., 2013) and GIFT (Gage et al., 2017) trials. However, in both trials genotype-guided dosing performed poorly or worse compared to clinically-guided dosing (COAG mean difference −8.3, 95% CI -15.0 to −2.0, *p* = 0.01, *n* = 255 patients; GIFT mean difference 0.2, 95% CI −8.9 to 9.4, *p* = 0.96, *n* = 102 patients), which has been attributed to the use of dosing algorithms that do not account for Black-African specific genetic variants. Importantly, no RCTs assessing the clinical utility of pharmacogenomic-guided dosing have recruited patients from sub-Saharan Africa or Latin-America.

### 4.5 Mixed/Other

Five RCTs recruiting from Sweden, United Kingdom and United States recruited a total of 29 (0.3% of 10,046) patients who were categorized as mixed/other/unknown, which means that categories that don’t fall under ‘White’, ‘Asian’, or ‘Black’ are not represented in warfarin pharmacogenomics clinical utility evidence.

## 5 Cost-Effectiveness Studies

Zhu and others have recently conducted a systematic review on the cost-effectiveness of pharmacogenomics-guided treatment for cardiovascular diseases, and included 16 studies on warfarin (Zhu et al., 2020a). In terms of representativeness, 14 trials reported the country/location including United States/Canada (*n* = 8, 57%), United Kingdom/Europe (*n* = 4, 29%) and Asia (*n* = 2, 14%) (Zhu et al., 2020a). Additionally, the two studies (You et al., 2004; You et al., 2009) that did not report the country were likely to have referred to the United States/Canada based on other publications (You et al., 2012; You, 2014) by the same authors. Eleven of the primary studies made reference to pharmacogenomic dosing algorithms including the IWPC algorithm (International Warfarin Pharmacogenetics Consortium et al., 2009) with or without other equations (*n* = 5), the Anderson/Carlquist equation (Carlquist et al., 2006) (*n* = 3), and the Caraco (Caraco et al., 2008), Gage (Gage et al., 2004), and Kim (Kim et al., 2009) equations (*n* = 1, for each). Except for one algorithm (Kim et al., 2009) that used only an Asian population and additional *CYP2C9* variants (*13, *14), the other algorithms used mainly White populations and included only CYP2C9*2 and CYP2C9*3, which means most current cost-effectiveness studies are mainly applicable to Whites.

## 6 Clinical Implementation Guidelines

Clinical implementation guidelines are key to transitioning pharmacogenomic knowledge into clinical practice (Abdullah-Koolmees et al., 2020). Accumulating evidence from observational studies and early randomized controlled trials such as (Hillman et al., 2005) prompted the United States Foods and Drug Administration (FDA) to update warfarin’s drug label (to include information on the relationship between genetic polymorphisms and warfarin doses) in August 2007 (Roper et al., 2010). However, professional organizations such as the American College of Medical Genetics and the American College of Chest Physicians demanded that further clinical research be undertaken before evidence-based guidelines could be generated (Roper et al., 2010). Prompted by further evidence, FDA further updated warfarin’s drug label in 2010 and included specific instructions on how to use the genotype information when predicting individualized doses (Finkelman et al., 2011). A year later, the Clinical Pharmacogenetics Implementation Consortium also published clinical practice recommendations to recommend warfarin dosing based on known *VKORC1/CYP2C9* genotypes (Johnson et al., 2011). As of 19 June 2020, four evidence-based pharmacogenomics clinical implementation guidelines currently exist (Abdullah-Koolmees et al., 2020), including the Dutch Pharmacogenetics Working Group (DPWG), the Clinical Pharmacogenetics Implementation Consortium (CPIC), the Canadian Pharmacogenomics Network for Drug Safety (CPNDS), and the French National Network (Réseau) of Pharmacogenetics (RNPGx). These guidelines are summarized in Table 3, with the discussion below again focusing on ethnic diversity.

**TABLE 3:** Clinical implementation guidelines (Abdullah-Koolmees et al., 2020).

Guideline	Genetic variants^a^	Algorithms	Evidence classification^b^
CPIC (Johnson et al., 2017)	Non-Africans: CYP2C9*2, CYP2C9*3, *VKORC1 -1639G > A*	Gage and IWPC equations (Gage et al., 2008; International Warfarin Pharmacogenetics Consortium et al., 2009)	Strong
	African: CYP2C9*2, CYP2C9*3, *VKORC1 -1639G > A*		Moderate
	African carriers of CYP2C9*5, *6, *8 or *11	Decrease calculated dose by 15–30% (20–40% in variant homozygotes)	Moderate
	African carriers of the *CYP2C* rs12777823 A allele	Decrease calculated dose by 10–25%	Moderate
CPNDS (Shaw et al., 2015)	CYP2C9*2, CYP2C9*3, *VKORC1 -1639G > A*	www.warfarindosing.org^c^	++++, Moderate
DPWG (The Dutch Pharmacogenetics Working Group, 2016)	CYP2C9*2, CYP2C9*3, *VKORC1 -1639G > A*	EU-PACT algorithms (IWPC equation as the key algorithm)	4A-D
RNPGx (Lamoureux et al., 2017)	CYP2C9*2, CYP2C9*3, *VKORC1 -1639G > A*	As per dosing table	Advisable

a For the CPIC, guidelines, optional variants such as CYP4F2*3 not included.

b CPIC, has three recommendation levels for genotype/phenotype-drug pairs (strong, moderate, and optional); CPNDS, has four levels of evidence (+ to ++++), and three levels for genotyping recommendations (strong, moderate, and optional); DPWG, has five (0–4) levels of evidence and eight for clinical relevance (AA, to F); and, RNPGx, has three levels for genotyping recommendations (essential, advisable, and possibly helpful).

c The primary algorithm is Gage 2008 (Gage et al., 2008). Nevertheless, many other studies (Millican et al., 2007; Lenzini et al., 2008; International Warfarin Pharmacogenetics Consortium et al., 2009; King et al., 2010) have contributed to the data and algorithms available at this site. For this reason, warfarindosing.org can also incorporate CYP4F2*3, *GGCX*, rs11676382 and additional *CYP2C9* alleles (CYP2C9*5 and CYP2C9*6). The IWPC, algorithm is also available on this site as a secondary algorithm.

CPIC, Clinical Pharmacogenetics Implementation Consortium; CPNDS, Canadian Pharmacogenomics Network for Drug Safety; DPWG, Dutch Pharmacogenetics Working Group; EU-PACT, European Pharmacogenetics of Anticoagulant Therapy; IWPC, International Warfarin Pharmacogenetics Consortium; RNPGx, French National Network (Réseau) of Pharmacogenetics.

From Table 3, the two key algorithms, that provide very similar dose recommendations (Johnson et al., 2017), are the Gage and IWPC equations which were both developed using data from large, predominantly White cohorts (Gage et al., 2008; International Warfarin Pharmacogenetics Consortium et al., 2009). Specifically, the Gage equation was developed from 1,015 warfarin-treated patients (83% White, 15% Black and 2% Mixed/Other) while the IWPC algorithm was developed from 4,043 patients (55% White, 30% Asian, 9% Black and 6% Mixed/Other), with the latter coming from nine countries across four continents. In our previous review, these were the most externally validated (Gage: 46 external validations; IWPC: 72 external validations) and clinically-assessed (Gage: eight clinical utility assessements including non-randomized studies; IWPC: seven clinical utility assessements) algorithms, a reason we also recommended them (Asiimwe et al., 2021). From Table 2, out of 10,046 randomized patients, at least 7,177 (71%) patients were recruited into 10 RCTs that evaluated one of these two algorithms, which again confirms their wide-spread use. However, out of these 1,177 patients, most patients either identified as White (*n* = 4,236, 45%) or Asian (*n* = 7,511, 49%), with only 416 (6%) and 14 (0.2%) patients identifying as Black (mostly African American) and of Mixed/Other ancestries respectively. When Hispanics are counted separately, they represent only 107 (<2%) of these patients.

Of the Gage and IWPC algorithms, only the Gage algorithm (warfarindosing.org) considers variants additional to CYP2C9*2, CYP2C9*3 and *VKORC1 -1639G > A* (Table 3) although it still misses out key variants such as CYP2C9*8, CYP2C9*11, and *CYP2C* rs12777823 that are likely to be of importance to Black patients and some Hispanic populations. For that reason, the CPIC guideline further recommends that calculated doses are further decreased for African patients who have these variants, although these recommendations are based on data derived primarily from African Americans, who are mostly of West African ancestry. For African Americans with only CYP2C9*2 and CYP2C9*3 genotype results, pharmacogenomic dosing is not recommended. Lastly, the CPIC guidelines also provide recommendations for paediatric patients, but these guidelines (of ‘moderate’ evidence rating) are applicable to only those with European ancestry (Johnson et al., 2017).

## 7 Future Directions in Warfarin Pharmacogenomic Research

### 7.1 Accounting for Race/Ethnicity in Warfarin Pharmacogenomic Research

When developing pharmacogenomic dosing algorithms, different races/ethnicities can be accounted for during analysis either through race/ethnic adjustment or stratification, with available evidence suggesting that race/ethnic stratification is superior in terms of better dose prediction (Shendre et al., 2018). In addition to an adequate representation of the different races/ethnicities in terms of trial sample sizes (Davis and Limdi, 2021), both race/ethnic adjustment and stratification also require that race/ethnicity is determined accurately and consistently across studies–the latter being important if different studies are to be compared. More advanced statistical techniques that can accurately and consistently infer genetic ancestry are currently available; however, genetic ancestry should not be viewed as a replacement for race/ethnic classifications as the latter are still important in biomedical research. As Borrell and others discuss, unlike genetic ancestry, race/ethnic classifications might be able to capture nongenetic causes of health inequities including those not captured by socioeconomic factors (Borrell et al., 2021). Although some organizations such as the United States National Institute of Health have previously defined several racial/ethnic categories for researchers to use, these categories remain broad and do not account for the heterogeneity within (and overlap/admixture between) the categories, which could lead to inconsistencies in race/ethnic categorization and overgeneralization of results which could negatively impact downstream clinical implementation (Zhang and Finkelstein, 2019). To be able to capture the many facets and constructs of race/ethnicity, including the social determinants of health and the biological/genetic determinants of drug response, more accurate measurements of racial/ethnic identity and evidence-based guidelines on the optimal use of self-identified race/ethnicity therefore need to be developed (Zhang and Finkelstein, 2019; Borrell et al., 2021).

### 7.2 Identifying Novel Genetic Variants in Underrepresented Populations

Whereas pharmacogenomic algorithms that incorporate both genetic and non-genetic factors can account for about half of the variability in warfarin daily dose requirements in Whites (Table 1), they explain only about a third in Blacks, meaning that Blacks and other underrepresented populations could benefit from more studies to identify additional genetic variants. Although the majority of previous association studies used a candidate-gene approach, genome-wide association studies (GWASs) (Cooper et al., 2008; Takeuchi et al., 2009; Cha et al., 2010; Perera et al., 2013; Parra et al., 2015; El Rouby et al., 2021) which systematically search the entire genome for new genetic factors have also been undertaken. One study in particular (Perera et al., 2013) was able to identify a new genetic variant (*CYP2C* rs12777823) that significantly altered dose requirements independent of the effects of CYP2C9*2, CYP2C9*3, and *VKORC1 -1639G > A*. The effects of this variant are unique to Africans, which implies that it would not have been discovered had an African [American] study not been conducted. Additionally and due to the high levels of haplotype diversity [low linkage disequilibrium (LD) levels], research conducted in African populations can benefit the more homogeneous Europeans/Asians through a finer mapping of the causal variants (Teo et al., 2010). This finer mapping will require that the causal variant be captured during genotyping/imputation, which means that genotyping panels (or reference panels used during imputation) should be customized for these populations.

GWASs are straightforward for more homogeneous populations but become more complicated in admixed populations such as African Americans and Hispanics/Latinos. GWASs that include admixed populations need to account for correlation between genetic variants that exists both at a fine scale (single nucleotide polymorphisms (SNPs) LD found in the homogeneous ancestral populations] and a coarse scale (admixture LD attributable to chromosomal segments of distinct ancestry) (Pasaniuc et al., 2011). Recent methods such as Tractor are able to account for both SNP and admixture LD in admixed populations which results in increased power and improved localization of GWAS signals (Pasaniuc et al., 2011; Atkinson et al., 2021).

Functional studies should be conducted for any novel genetic variants to validate the gene-drug response. These should be complemented by validating the same response across populations, and in the absence of functional data, studies in other populations could help rule out causality as was demonstrated for *CYP2C* rs12777823 (Perera et al., 2013).

Lastly, the possibility that response to warfarin treatment in some of the populations may be polygenic (influenced by a large number of genetic variants with small effects, rather than a few genetic variants with large effects) (Lewis and Vassos, 2020) should not be ruled out, and where it is not possible to identify a few variants that can greatly influence warfarin response, polygenic risk scores or dosing models should be considered.

### 7.3 Improving Clinical Utility Through Ethnic Diversity

The failure to account for all ethnicities in warfarin pharmacogenomic algorithms could be one of the reasons some studies have reported low clinical utilities. For example, the COAG trial (Kimmel et al., 2013) reported that pharmacogenomic-guided dosing did not improve the PTTR when compared to clinically-guided dosing (mean PTTR decreased by 0.2, *p* = 0.91, *n* = 955). However, this could be attributed to including a relatively large proportion of African Americans (27%), who did not benefit from using an algorithm that did not consider African-specific genetic variants [mean PTTR decreased by 8.3, which was statistically significant (*p* = 0.01)]. When non-Blacks were analysed in the COAG trial, the treatment effect changed direction (mean TTR increased by 2.8%) although it remained non-significant (*p* = 0.15), which was probably due to a decreased sample size (*n* = 700) for this treatment effect. Indeed, when a later trial (GIFT) analysed more patients (*n* = 1,588) and included fewer Blacks (6%), genotype-guided dosing increased the mean PTTR by 3.4, which was statistically significant (*p* = 0.004) (Gage et al., 2017). Going forward, if all races/ethnicities can be accounted for in warfarin dosing algorithms, there should be less discordance in RCTs evaluating their clinical utility, and together with the decreasing costs of genotyping lead to more cost-effectiveness studies that are in support of genotype-guided dosing. This evidence should lead to clinical implementation guidelines that cater for more ethnicities and should also make it easier to obtain endorsement from all stakeholders such as patients, healthcare professionals, and healthcare payers.

### 7.4 Reducing Healthcare Disparities Through Ethnic Diversity

As the COAG trial (Kimmel et al., 2013) demonstrated, using pharmacogenomic dosing algorithms that do not include ethno-specific genetic factors can lead to sub-optimal anticoagulation for these populations, which could exacerbate healthcare disparities (Martin et al., 2017). For example, in previous work we have conducted in sub-Saharan Africa (South Africa and Uganda), we observed that fixed-dose initiation was associated with a median time in therapeutic range (TTR) of 41% (Semakula et al., 2020), which is much lower than that observed in an European (Sweden and United Kingdom) cohort (mean TTR 60%) (Pirmohamed et al., 2013). Implementing a pharmacogenomic-dosing algorithm in the European cohort increased the mean TTR to 67% (Pirmohamed et al., 2013), which makes the anticoagulation quality gap with sub-Saharan Africa even wider as there is no validated pharmacogenomic dosing algorithm in this region at the moment (Mouton et al., 2021). Moreover, Europe which is wealthier than sub-Saharan Africa is able to afford the more costly new direct oral anticoagulants (DOACs), whereas sub-Saharan Africa’s choices are mainly limited to warfarin due to its low cost (Semakula et al., 2021). Because it is important that the quality of warfarin anticoagulation is optimal for all populations [warfarin is equal to DOACs in terms of safety and efficacy if it is given to patients who are able to maintain a time in therapeutic INR range ≥66% (Ruff et al., 2014)], we are currently developing a pharmacogenomic dosing algorithm for the two sub-Saharan countries and validating a point-of-care test that includes African-specific genetic variants in an ongoing collaborative project (War-PATH, Warfarin anticoagulation in PATients in sub-SaHaran Africa; http://warpath.info/). We call upon other researchers to conduct similar research in other underrepresented populations. Journal editors and reviewers (both grants and manuscripts) need to also recognize the importance of pharmacogenomic research in underrepresented populations and should not consider studies published in minority populations to be less important/novel simply because similar research has been performed in Whites or Asians (Perera et al., 2014).

### 7.5 Impact of DOACs on Warfarin Pharmacogenomic Research

DOACs are gradually replacing oral vitamin K antagonists (VKAs) such as warfarin. For example, whereas warfarin remains the most frequently prescribed individual oral anticoagulant in England (38% of oral anticoagulant prescriptions in 2019), the proportion of DOAC (apixaban, dabigatran, edoxaban, or rivaroxaban) prescriptions increased from 16% in 2015 to 62% in 2019 (Afzal et al., 2021). Due to non-inferior and/or superior outcomes in non-valvular atrial fibrillation patients, many clinical guidelines have recommended DOAC use with the World Health Organization recently including DOACs on its 21st Model List of Essential Medicines, which should improve accessibility in resource-limited settings (Zaidel et al., 2020). However, and even with the recent approval of cheaper DOAC generics (e.g. apixaban was approved in the United States in 2019 and rivaroxaban in Europe in 2020), these are likely to remain more expensive than generic warfarin and it will be some time before they are available in low income countries such as those in sub-Saharan Africa (Neumann et al., 2021; Noubiap and Kamtchum-Tatuene, 2022). Consequently, warfarin pharmacogenomic research is likely to remain important for underrepresented groups for whom warfarin is more readily available and/or affordable.

Additionally, and despite most key trials being multinational (Lee et al., 2021), the evidence supporting the use of DOACs over warfarin is mostly from Whites (Davis and Limdi, 2021). For example, the ROCKET AF (Rivaroxaban Once Daily Oral Direct Factor Xa Inhibition Compared with Vitamin K Antagonism for Prevention of Stroke and Embolism Trial in Atrial Fibrillation) trial recruited from 1,178 centres (45 countries) a total of 13,997 participants, with the majority (83%) being White (other categories were 13% Asians, 1% Black and 3% other) (Piccini et al., 2014; Lee et al., 2021). This means that this evidence may be less applicable to underrepresented populations. Specifically, rivaroxaban may not be safer than warfarin in sub-Saharan Africa as many sub-Saharan patients also receive P-glycoprotein and *CYP3A4*-modulating drugs for tuberculosis and HIV, drugs which have been reported to attenuate rivaroxaban’s advantage in terms of major bleeding risk (Harskamp et al., 2019; Noubiap and Kamtchum-Tatuene, 2022). The safety and efficacy of DOACs should therefore be appropriately assessed in the underrepresented populations before recommendations regarding their replacement of warfarin are made for these populations. This is important as it is now recognized that due to genetic (such as variants found in the genes *CES1*, *ABCB1*, *CYP3A4*, and *CYP3A5*) and non-genetic (such as renal function and drug-drug interactions) factors, DOACs also have significant interindividual variability in dose response, which can lead to haemorrhagic or thrombotic events in particular groups of patients, although the literature is scare and more studies are still required (Lahteenmaki et al., 2021; Raymond et al., 2021).

It should also be pointed out that only a limited number of studies have compared the efficacy of DOACs versus genotype-guided warfarin treatment. This is important since the comparative advantages of DOACs may be greater in patients with certain warfarin-related genotypes. For example, in a subgroup analysis of the ENGAGE AF-TIMI 48 (Effective aNticoaGulation with factor xA next GEneration in Atrial Fibrillation-Thrombolysis In Myocardial Infarction study 48) trial, of the 4,833 patients taking warfarin (dosed based on the patient’s clinical profile), 1711 (34%) and 140 (3%) respectively were categorized as sensitive and highly sensitive responders depending on their *CYP2C9* and *VKORC1* genotypes (Mega et al., 2015). Compared with the “normal responders” on warfarin, edoxaban reduced bleeding in the sensitive and highly sensitive responder groups (60 mg edoxaban *P*-interaction = 0·0066; 30 mg edoxaban *P*-interaction = 0·0036) in the first 90 days. Thus, the sensitive and highly sensitive responder groups seemed to derive greater safety benefit from edoxaban in terms of fewer bleeding episodes. Since genotype-guided warfarin dosing is beneficial in terms of reducing the number of bleeding episodes (Tse et al., 2018; Wang et al., 2022), especially for the sensitive and highly sensitive responders, whether DOACs would show superiority in terms of both efficacy and safety against genotype-guided warfarin dosing is unclear. This is a question that is unlikely to be answered as it would require a large randomised controlled trial, which would be expensive and unlikely to be funded from academic or industrial sources.

DOACs are partly eliminated by the kidneys, and can therefore accumulate in patients with renal impairment, which increases the risk of bleeding (Lutz et al., 2017). It is therefore advisable that DOACs be avoided in patients with an estimated glomerular filtration rate (eGFR) below 30 ml/min, especially for the DOACs with high rates of renal elimination. For patients with mild/moderate renal impairment, caution is advised when using DOACs as renal function can deteriorate abruptly which necessitates regular renal function (eGFR) monitoring (Lutz et al., 2017). Compared to Whites, the risk of using DOACs in renally-impaired patients may be more pronounced in underrepresented populations such as Blacks, who are more likely to develop end-stage kidney failure (for which DOACs are contraindicated) and for whom eGFR-estimating equations are less accurate (making renal function monitoring less reliable) (Powe, 2020). In addition to patients with severe renal impairment, warfarin remains the only choice for those with heart valves, children, and patients on certain medications (Burn and Pirmohamed, 2018). Lastly, the lack of a reliable biomarker (makes it hard to monitor treatment), twice daily dosing regimens for some DOACs like apixaban and dabigatran (may lead to poor adherence), and costly antidotes such as idarucizumab and andexanet alfa (makes reversal of overdosing expensive) (Burn and Pirmohamed, 2018; Godier and Martin, 2019) means that warfarin may remain the anticoagulant of choice for many underrepresented populations, which makes it important to continue conducting warfarin pharmacogenomic research in these groups.

### 7.6 Ethnic Diversity and Pharmacogenomics: Warfarin as a Pathfinder Drug

Being regarded as a poster child for pharmacogenomics (Pirmohamed, 2014), warfarin has previously set both the trends and directions in pharmacogenomic research. It is therefore not surprising that considerably fewer pharmacogenomic studies have been published for other vitamin K antagonists when compared with warfarin. For example, only five and two dosing algorithms were previously reported for acenocoumarol and phenprocoumon, respectively, compared with 32 for warfarin (Verhoef et al., 2014). The seven acenocoumarol and phenprocoumon pharmacogenetic dosing algorithms were developed in European (Germany, Greece, Netherlands, Spain) and Asian (India) countries which means, like warfarin, they do not reflect ethnic diversity. In the broader cardiovascular field, clopidogrel and statins are other drugs that are currently ready for pharmacogenomic implementation (Magavern et al., 2021), and are discussed further below.

Clopidogrel is a thienopyridine pro-drug that is metabolized in the liver to an active metabolite that inhibits platelet aggregation (Lamoureux et al., 2017; Lee et al., 2022). The key metabolizing enzyme is Cytochrome P450 isoform 2C19 (CYP2C19), coded for by the *CYP2C19* gene, with null function alleles such as CYP2C19*2 (rs4244285, splicing defect with appearance of a stop codon) and CYP2C19*3 (rs4986893, nonsense variant with a premature stop codon) being associated with slow metabolizer phenotypes (reduced therapeutic efficacy/poor response to clopidogrel as less clopidogrel is transformed to its active metabolite). On the other hand, CYP2C19*17 (rs12248560, gene promoter variant) increases gene transcription and protein expression which can lead to excessive inhibition of platelet aggregation and increased risk of haemorrhagic events in clopidogrel-treated carriers of this allele (Lamoureux et al., 2017; Lee et al., 2022). Due to differences in MAFs for these SNPs [CYP2C19*2 African = 0.17, American = 0.11, East Asian = 0.31, European = 0.15, South Asian = 0.36; CYP2C19*3 East Asian = 0.06, South Asian = 0.01, Other ∼0.00; and, CYP2C19*17 African = 0.24, American = 0.12, East Asian = 0.02, European = 0.22, South Asian = 0.14 (Genomes Project et al., 2015)], the importance of these *CYP2C19* variants will differ from population to population, which also means that studies conducted in one population may not be applicable to another. However, most pharmacogenomics research evaluating the association between *CYP2C19* and antiplatelet therapy (mostly clopidogrel) has been conducted in Whites (Davis and Limdi, 2021), which doesn’t reflect ethnic diversity and which could lead to clinical or other consequences. For example, the clopidogrel manufacturer was sued for not informing Hawaiians that clopidogrel may be ineffective due to the higher prevalence of CYP2C19*2 and CYP2C19*3 in Hawaii compared to Whites who comprised 95% of the CAPRIE (clopidogrel versus aspirin in patients at risk of ischaemic events) study (*n* = 19,185) that was pivotal to clopidogrel’s approval in the United States (Wu et al., 2015).

Statins such as atorvastatin and simvastatin inhibit 3-hydroxyl-3-methylglutaryl coenzyme A (HMB-CoA) reductase thereby inhibiting a key step in cholesterol synthesis (the transformation of HMG-CoA into mevalonate) (Lamoureux et al., 2017). The key gene involved in statin pharmacokinetics is solute carrier organic anion transporter family member 1B1 (*SLC O 1B1*) which encodes the SLCO1B1 protein (also known as organic anion transporting polypeptide family member 1B1 [OATP1B1] and which captures the statins for clearance through hepatobiliary excretion). The key variant is SLC O 1B1*5 (rs4149056, nonsense variant) which is associated with decreased enzyme transporter activity and decreased hepatobiliary excretion of statins–this increases serum concentrations increasing susceptibility to muscle toxicity that ranges from simple myalgia to potentially lethal rhabdomyolysis, depending on the genotype (heterozygous vs. homozygous carriers), statin dose, and statin type (simvastatin being the most implicated of currently approved statins) (Ramsey et al., 2014; Lamoureux et al., 2017). Differences in MAFs for this variant have also been observed being 0.01, 0.13, 0.12, 0.16 and 0.04 for Africans, Americans, East Asians, Europeans, and South Asians respectively (Genomes Project et al., 2015). Compared to warfarin and clopidogrel, pharmacogenomics studies for statins are much fewer (Magavern et al., 2021), and they are also likely to be more applicable to Whites. For example, in the most recently reported statin pharmacogenomics RCT, 86% of the 408 recruited patients were White (Vassy et al., 2020) while two recent large-scale GWASs that analysed a total of more than 20,000 patients included only those of European descent (Carr et al., 2019; Trompet et al., 2021).

By ensuring that warfarin pharmacogenomic research is ethnically diverse, it is expected that other pharmacogenomic fields will follow suit, as warfarin is a pathfinder drug in pharmacogenomic research.

## 8 Summary

In conclusion, most of the current warfarin pharmacogenomic research, in particular clinical utility studies, is applicable to Whites and Han-Chinese, which means other races/ethnicities including many non-Chinese Asians, Blacks, Hispanics/Latinos, American Indians/Alaska Natives and Native Hawaiians/other Pacific Islanders are either underrepresented or not represented in the evidence base. There are key differences in the minor allele frequencies of the genetic factors that influence warfarin response between these populations which means that pharmacogenomic dosing algorithms that work well in one population may perform poorly in another. Due to the availability of sufficient evidence from both observational and randomized clinical trials, clinical implementation guidelines for warfarin dosing have been developed although these are mostly applicable to White populations. This means that existing pharmacogenomics evidence does not reflect diversity, which is likely to exacerbate health inequalities. Further pharmacogenomic research which is focused on underrepresented populations is recommended in order to ensure that warfarin pharmacogenomics achieves its full potential for all races/ethnicities and health inequalities are reduced.
