# Supplementary material for: Ethnic Diversity and Warfarin Pharmacogenomics
Source: Front Pharmacol. 2022 Apr 4;13:866058. doi: 10.3389/fphar.2022.866058 (PMC9014219; doi:10.3389/fphar.2022.866058)
Supplement: Supplementary file 1 [file Table1.DOCX]

**Supplementary Table S1.** **Genes involved in warfarin’s mechanism of action.**

| **Pathway (Wadelius et al., 2007;Wadelius and Pirmohamed, 2007)** | **Gene (NCBI gene ID) (National Center for Biotechnology Information, 2018)** | **Protein function**  **(NCBI, (National Center for Biotechnology Information, 2018) unless otherwise)** | **Examples of SNPs (unless shown otherwise, examples are from (Wadelius et al., 2007))** | | | | |
| --- | --- | --- | --- | --- | --- | --- | --- |
|  |  |  | **Reference SNP ID (National Center for Biotechnology Information, 2018)** | **Location** | **Effects** | | **MAF (1000’s genome project) (European Bioinformatics lnstitute, 2018)** |
|  |  |  |  |  | **Functional** | **On warfarin dosing** |  |
| **Vitamin K Biotransformation** | | | | | | | |
| *Vitamin K cycle* | | | | | | | |
| Vitamin K epoxide reductase | *VKORC1* (79001) | A hepatic epoxide hydrolase that catalyses the reduction of vitamin K 2,3-epoxide. It is warfarin’s molecular target. | rs9923231  (*G3673A* or –*1639G>A*) | Promoter region (5' UTR) | Alters a *VKORC1* transcription factor binding site (E-Box, consensus sequence *CANNTG* that may function as a repressor binding site, in the 5’ UTR). An *A>G* mutation abolishes the E-box consensus increasing the promoter activity by 44% (Yuan et al., 2005). The reverse *G>A* mutation re-instates the consensus leading to reduced transcription/amounts of mRNA and fewer functional copies of the VKORC1 protein (Rieder et al., 2005;Yuan et al., 2005). This mechanism is yet to be confirmed. This SNP is in near perfect LD with others e.g. rs9934438 below (Daly, 2013). | The *A* allele is associated with a low-dose (reduced doses up to 3 mg/day) (Jorgensen et al., 2012). It is the most important predictor of warfarin dose explaining ~27% of the variance in warfarin maintenance dose in Caucasians and Asians (only ~9% of dose variability in African Americans is explained, attributable to a lower allele frequency) (Asiimwe et al., 2021). | AFR = 0.05  AMR = 0.41  EAS = 0.89  EUR = 0.39  SAS = 0.15 |
|  |  |  | rs9934438  (*C6484T* or *1173C>T*) | Intronic | Unknown function (D'Andrea et al., 2005). | First SNP to be associated with a low-dose warfarin phenotype (D'Andrea et al., 2005). | AFR = 0.05  AMR = 0.41  EAS = 0.89  EUR = 0.39  SAS = 0.15 |
| Epoxide hydrolase 1, microsomal | *EPHX1* (2052) | Biotransformation enzyme (Pautas et al., 2010;Ciccacci et al., 2011). | rs1051740 | Coding | Unknown function (Loebstein et al., 2005). | Likely to increase warfarin dose requirements (Loebstein et al., 2005;Schelleman et al., 2010). | AFR = 0.14  AMR = 0.32  EAS = 0.48  EUR = 0.30  SAS = 0.38 |
| NAD(P)H dehydrogenase,  quinone 1 | *NQO1* (1728) | A detoxifying enzyme that can reduce the vitamin K quinone (Wallin and Hutson, 1982;Ross and Siegel, 2004). | rs1437135 | Intronic | Associated with protein C levels (Buil et al., 2004). | Lower protein C levels increase thrombotic risk which may necessitate increased warfarin doses. | AFR = 0.26  AMR = 0.34  EAS = 0.42  EUR = 0.21  SAS = 0.36 |
| Calumenin | *CALU* (813) | Inhibits GGCX | rs339097 | Intronic | Associated with higher *CALU* expression although it remains unclear if affects mRNA expression or stability or is in LD with causative SNP(s) (Voora et al., 2010). | Associated with higher warfarin dose requirements in African Americans (Voora et al., 2010;Ramirez et al., 2012). | AFR = 0.14  AMR = 0.01  EAS = 0.01  EUR = 0.00  SAS = 0.01 |
| Gamma-glutamyl  carboxylase | *GGCX* (2677) | Carboxylates vitamin K dependent coagulation factors/proteins (Rost et al., 2004). | rs699664 | Coding | Unknown function (Loebstein et al., 2005). | Associated with higher dose in European and Asian patients (Wadelius et al., 2005;Kimura et al., 2007;Huang et al., 2011;Kamali et al., 2013) but not in African Americans (Schelleman et al., 2010;Cavallari et al., 2012;Ramirez et al., 2012). | AFR = 0.663  AMR = 0.269  EAS = 0.310  EUR = 0.367  SAS = 0.151 |

**Supplementary Table S1. Continued.**

| **Pathway (Wadelius et al., 2007;Wadelius and Pirmohamed, 2007)** | **Gene (NCBI gene ID) (National Center for Biotechnology Information, 2018)** | **Protein function**  **(NCBI, (National Center for Biotechnology Information, 2018) unless otherwise)** | **Examples of SNPs (unless shown otherwise, examples are from (Wadelius et al., 2007))** | | | | |
| --- | --- | --- | --- | --- | --- | --- | --- |
|  |  |  | **Reference SNP ID (National Center for Biotechnology Information, 2018)** | **Location** | **Effects** | | **MAF (1000’s genome project) (European Bioinformatics lnstitute, 2018)** |
|  |  |  |  |  | **Functional** | **On warfarin dosing** |  |
| *Vitamin K metabolism* | | | | | | | |
| Cytochrome P450 4F2 | *CYP4F2* (8592) | A vitamin K oxidase that catalyzes the metabolism of vitamin K to hydroxyl-vitamin K1 (Caldwell et al., 2008). | rs2108622  (*1297G>A, CYP4F2*3* or V433M) | Coding | Encodes a protein with decreased activity hence increased vitamin K levels and warfarin resistance (Caldwell et al., 2008;McDonald et al., 2009). | Higher warfarin dose requirements in Caucasians and Asians (Caldwell et al., 2008;Takeuchi et al., 2009;Cha et al., 2010;Liang et al., 2012;Johnson and Cavallari, 2015) but not in African Americans (Perera et al., 2013;Shendre et al., 2016). It however explains only an additional 1–2% of observed warfarin dose variability in Caucasians/Asians (Takeuchi et al., 2009;Cha et al., 2010). | AFR = 0.08  AMR = 0.24  EAS = 0.21  EUR = 0.29  SAS = 0.41 |
| *Vitamin K-dependent proteins (other genes not detailed include: F9 (gene ID 2158), F10 (2159), PROS1 (5627), and GAS6 (2621))* | | | | | | | |
| Coagulation factor II, prothrombin | *F2* (2147) | Converts fibrinogen to fibrin, activates FV, FVIII, FXIII and protein C (Berkner, 2000;Dahlback, 2005). | rs5896 | Coding | Function unknown. | May increase warfarin sensitivity (D'Ambrosio et al., 2004;Shikata et al., 2004). | AFR = 0.01  AMR = 0.28  EAS = 0.60  EUR = 0.12  SAS = 0.17 |
| Coagulation factor VII | *F7* (2155) | FVIIa converts FIX to FIXa and FX to FXa (Berkner, 2000;Dahlback, 2005). | rs6046 | Coding | Associated with reduced concentration and activity of the active protein (Arbini et al., 1994;Mlynarsky et al., 2012). | Associated with lower warfarin doses in Israelites (Mlynarsky et al., 2012). | AFR = 0.12  AMR = 0.12  EAS = 0.05  EUR = 0.11  SAS = 0.30 |
| Protein C | *PROC* (5624) | Activated protein C inactivates FVa and VIIIa (Berkner, 2000;Dahlback, 2005). | rs1799809 | Regulatory region | Lower protein C activity (Spek et al., 1995;Aiach et al., 1999). | Lower protein C levels increase thrombotic risk which may necessitate increased warfarin doses. | AFR = 0.26  AMR = 0.71  EAS = 0.82  EUR = 0.59  SAS = 0.62 |
| Protein Z | *PROZ* (8858) | Is a cofactor for the inactivation of FXa (Berkner, 2000;Broze, 2001). | rs3024711 | Intron | Function unknown. | Unclear effects. | AFR = 0.06  AMR = 0.22  EAS = 0.30  EUR = 0.17  SAS = 0.44 |
| *Other coagulation proteins (another gene example not detailed is SERPINC1, ID 462)* | | | | | | | |
| Coagulation factor V | *F5* (2153) | A cofactor that activates FII and FXa. | rs6025 | Coding | FV Leiden increased risk of thrombosis (Bertina et al., 1994;Dahlback, 2005). | Increased thrombotic risk implies increased warfarin dose requirements. | AFR = 0.00  AMR = 0.01  EAS = 0.00  EUR = 0.01  SAS = 0.01 |

**Supplementary Table S1. Continued.**

| **Pathway (Wadelius et al., 2007;Wadelius and Pirmohamed, 2007)** | **Gene (NCBI gene ID) (National Center for Biotechnology Information, 2018)** | **Protein function**  **(NCBI, (National Center for Biotechnology Information, 2018) unless otherwise)** | **Examples of SNPs (unless shown otherwise, examples are from (Wadelius et al., 2007))** | | | | |
| --- | --- | --- | --- | --- | --- | --- | --- |
|  |  |  | **Reference SNP ID (National Center for Biotechnology Information, 2018)** | **Location** | **Effects** | | **MAF (1000’s genome project) (European Bioinformatics lnstitute, 2018)** |
|  |  |  |  |  | **Functional** | **On warfarin dosing** |  |
| *Transportation* | | | | | | | |
| Apolipoprotein E | *APOE* (348) | Serves as a ligand for vitamin K-uptake mediating receptors.(Berkner and Runge, 2004) | rs429358 | Coding | These 2 SNPs discriminate between the haplotypes ε2, ε3 and ε4 which are respectively associated with low, intermediate and high vitamin K uptake (Schelleman et al., 2007). | ε2ε2 genotype associated with lower warfarin doses than ε3ε3 or ε4ε4 (Yu et al., 2016). | AFR = 0.27  AMR = 0.10  EAS = 0.09  EUR = 0.16  SAS = 0.09 |
|  |  |  | rs7412 | Coding |  |  | AFR = 0.10  AMR = 0.05  EAS = 0.10  EUR = 0.06  SAS = 0.04 |
| ***Warfarin biotransformation*** | | | | | | | |
| *Metabolism (other genes not detailed include: CYP2C8 (ID 1558), CYP2C18 (1562), CYP2C19 (1557), CYP1A1 (1543), CYP1A2 (1544), CYP3A4 (1576), and CYP3A5 (1577)).* | | | | | | | |
| Cytochrome P450 2C9 | *CYP2C9* (1559) | Polymorphic hepatic drug metabolising enzyme (S-warfarin). | rs1799853 (*430C>T, R144C, *2*) | Coding | About 12% of wild-type activity (Rettie et al., 1994;Haining et al., 1996;Crespi and Miller, 1997). | Leads to a reduction in warfarin dose by up to 1.5 mg/day (Jorgensen et al., 2012). | AFR = 0.01  AMR = 0.10  EAS = 0.00  EUR = 0.12  SAS = 0.04 |
|  |  |  | rs1057910 (*1075A>C, I359L *3*) | Coding | <5% as efficient as the wild-type enzyme (Rettie et al., 1994;Haining et al., 1996;Sullivan-Klose et al., 1996;Crespi and Miller, 1997) . | Leads to reduction in dose by up to 2.6 mg/day (Jorgensen et al., 2012). | AFR = 0.00  AMR = 0.04  EAS = 0.03  EUR = 0.07  SAS = 0.11 |
|  |  |  | rs28371686 (*1080 C>G, D360E, *5*) | Coding | Reduced and null (*6) enzyme activity. | Leads to lower dose requirements, especially in populations of African ancestry (Asiimwe et al., 2020). Overall, *CYP2C9* genotype accounts for ~7–10% of warfarin dose variability (Johnson and Cavallari, 2013;Johnson et al., 2017). | AFR = 0.02  Others ~ 0.00 |
|  |  |  | rs9332131 (818delA, **6*) | Coding |  |  | AFR = 0.01  Others ~ 0.00 |
|  |  |  | rs7900194 (*449 G>A, R150H, *8*) | Coding |  |  | AFR = 0.05  Others ~ 0.00 |
|  |  |  | rs28371685 (1003 A>G, R335W, *11) | Coding |  |  | AFR = 0.02  Others ~ 0.00 |
|  |  |  | rs12777823 | (*CYP2C* gene cluster region) | Associated with alterations in warfarin clearance. May be in LD with another variant because the effect is observed only in African Americans (Perera et al., 2013). | Heterozygous or homozygous African Americans (A allele) respectively require a dose reduction of ~ 7 or 9 mg/week (Perera et al., 2013). | AFR = 0.251  AMR = 0.107  EAS = 0.314  EUR = 0.151  SAS = 0.362 |

**Supplementary Table S1. Continued.**

| **Pathway (Wadelius et al., 2007;Wadelius and Pirmohamed, 2007)** | **Gene (NCBI gene ID) (National Center for Biotechnology Information, 2018)** | **Protein function**  **(NCBI, (National Center for Biotechnology Information, 2018) unless otherwise)** | **Examples of SNPs (unless shown otherwise, examples are from (Wadelius et al., 2007))** | | | | |
| --- | --- | --- | --- | --- | --- | --- | --- |
|  |  |  | **Reference SNP ID (National Center for Biotechnology Information, 2018)** | **Location** | **Effects** | | **MAF (1000’s genome project) (European Bioinformatics lnstitute, 2018)** |
|  |  |  |  |  | **Functional** | **On warfarin dosing** |  |
| *Cytochrome P450 inducibility* | | | | | | | |
| Pregnane X receptor (PXR) | NR1I2 (8856) | Mediates induction of *CYP2C9*, other CYP enzymes and *ABCB1* (Chen et al., 2004). | rs2472682 | Intronic | Unknown function. | Variant homozygote carriers required significantly lower daily doses than wild-type homozygotes by about 0.8 mg. This SNP accounted for 2.3% of dose variability (Moon et al., 2015). | AFR = 0.15  AMR = 0.65  EAS = 0.38  EUR = 0.66  SAS = 0.55 |
| Constitutive androstane receptor (CAR) | NR1I3 (9970) | Transcriptional regulation of several genes e.g. *CYP2C9* (Assenat et al., 2004). | rs2501873 | Intronic | Unknown function. | Lower dosing. Accounted for 1.3% of variability in warfarin dose (Moon et al., 2015). | AFR = 0.31  AMR = 0.52  EAS = 0.43  EUR = 0.56  SAS = 0.52 |
| *Transportation (another gene not detailed is ORM2, ID 5005)* | | | | | | | |
| P-glycoprotein, Multidrg resistance protein 1 | *ABCB1* (5243) | A xenobiotics cellular efflux pump (Kroetz et al., 2003). | rs2032582 | Coding | Warfarin is a week inhibitor and maybe a substrate (Sussman et al., 2002). | Could theoretically increase dose requirements. | AFR = 0.00  AMR = 0.06  EAS = 0.13  EUR = 0.02  SAS = 0.05 |
| Alpha-1-acid glycolprotein 1, Oroso-cumoid 1 | *ORM1* (5004) | A warfarin carrier in plasma.(Nakagawa et al., 2003) | rs1687390 | Regulatory region | Unknown function. | Could decrease dose requirements. | AFR = 0.30  AMR = 0.07  EAS = 0.00 |

Abbreviations: *ABCB1*, P-glycoprotein gene or *MDR1* gene; AFR, African; AMR, American; *APOE*, Apolipoprotein E gene; *CALU*, Calumenin gene; *CAR*, Constitutive androstane receptor; *CYP*, Cytochrome P450; EAS, East Asian; *EPHX1*, Epoxide hydrolase 1, microsomal gene; EUR, European; *GAS6*, Growth-arrest specific 6; *GGCX*, Gamma-glutamyl carboxylase gene; *F2*, Coagulation factor II gene or prothrombin gene; *F5*, Coagulation factor V gene; *F7*, Coagulation factor VII gene; *F9*, Coagulation factor IX gene; *F10*, Coagulation factor X gene; *FII*, Coagulation factor II or prothrombin (additional a, if any, stands for activated); *FV*, Coagulation factor V; *FVII*, Coagulation factor VII; FIX, Coagulation factor IX; FX, Coagulation factor X; ID, identification; LD, linkage disequilibrium; *NQO1*, NAD(P)H dehydrogenase, quinone 1 gene; *MDR1*, Multidrug resistance protein 1; NCBI, U.S. National Center for Biotechnology Information; *NR1I2*, Pregnane X receptor gene; *NR1I3*, Constitutive androstane receptor gene; *ORM1*, Orosomucoid 1 gene or Alpha-1-acid glycoprotein 1 gene; *ORM2*, Orosomucoid 2 gene or Alpha-1-acid glycoprotein 2 gene; *PROC*, Protein C gene; *PROS1*, Protein S gene; *PROZ*, Protein Z gene; *PXR*, Pregnane X receptor; SAS, South Asian; *SERPINC1*, Anti-thrombin III gene; SNP, Single nucleotide polymorphism; UTR, Untranslated region; *VKOR*, vitamin K epoxide reductase complex.

**Supplementary References**

Aiach, M., Nicaud, V., Alhenc-Gelas, M., Gandrille, S., Arnaud, E., Amiral, J., Guize, L., Fiessinger, J.N., and Emmerich, J. (1999). Complex association of protein C gene promoter polymorphism with circulating protein C levels and thrombotic risk. *Arterioscler Thromb Vasc Biol* 19**,** 1573-1576.

Arbini, A.A., Bodkin, D., Lopaciuk, S., and Bauer, K.A. (1994). Molecular analysis of Polish patients with factor VII deficiency. *Blood* 84**,** 2214-2220.

Asiimwe, I.G., Zhang, E.J., Osanlou, R., Jorgensen, A.L., and Pirmohamed, M. (2021). Warfarin dosing algorithms: A systematic review. *British Journal of Clinical Pharmacology* 87**,** 1717.

Asiimwe, I.G., Zhang, E.J., Osanlou, R., Pirmohamed, M., Krause, A., Dillon, C., Beasly, M.T., Limdi, N.A., Suarez-Kurtz, G., Zhang, H., Perera, M.A., Perini, J.A., Renta, J.Y., Duconge, J., Cavallari, L.H., Marcatto, L.R., Santos, P.C.J.L., Kimmel, S.E., Lubitz, S.A., Scott, S.A., Kawai, V.K., and Jorgensen, A.L. (2020). Genetic Factors Influencing Warfarin Dose in Black-African Patients: A Systematic Review and Meta-Analysis. *Clinical Pharmacology and Therapeutics* 107**,** 1420-1433.

Assenat, E., Gerbal-Chaloin, S., Larrey, D., Saric, J., Fabre, J.M., Maurel, P., Vilarem, M.J., and Pascussi, J.M. (2004). Interleukin 1beta inhibits CAR-induced expression of hepatic genes involved in drug and bilirubin clearance. *Hepatology* 40**,** 951-960.

Berkner, K.L. (2000). The vitamin K-dependent carboxylase. *J Nutr* 130**,** 1877-1880.

Berkner, K.L., and Runge, K.W. (2004). The physiology of vitamin K nutriture and vitamin K-dependent protein function in atherosclerosis. *J Thromb Haemost* 2**,** 2118-2132.

Bertina, R.M., Koeleman, B.P., Koster, T., Rosendaal, F.R., Dirven, R.J., De Ronde, H., Van Der Velden, P.A., and Reitsma, P.H. (1994). Mutation in blood coagulation factor V associated with resistance to activated protein C. *Nature* 369**,** 64-67.

Broze, G.J., Jr. (2001). Protein Z-dependent regulation of coagulation. *Thromb Haemost* 86**,** 8-13.

Buil, A., Soria, J.M., Souto, J.C., Almasy, L., Lathrop, M., Blangero, J., and Fontcuberta, J. (2004). Protein C levels are regulated by a quantitative trait locus on chromosome 16: results from the Genetic Analysis of Idiopathic Thrombophilia (GAIT) Project. *Arterioscler Thromb Vasc Biol* 24**,** 1321-1325.

Caldwell, M.D., Awad, T., Johnson, J.A., Gage, B.F., Falkowski, M., Gardina, P., Hubbard, J., Turpaz, Y., Langaee, T.Y., Eby, C., King, C.R., Brower, A., Schmelzer, J.R., Glurich, I., Vidaillet, H.J., Yale, S.H., Qi Zhang, K., Berg, R.L., and Burmester, J.K. (2008). CYP4F2 genetic variant alters required warfarin dose. *Blood* 111**,** 4106-4112.

Cavallari, L.H., Perera, M., Wadelius, M., Deloukas, P., Taube, G., Patel, S.R., Aquino-Michaels, K., Viana, M.A., Shapiro, N.L., and Nutescu, E.A. (2012). Association of the GGCX (CAA)16/17 repeat polymorphism with higher warfarin dose requirements in African Americans. *Pharmacogenet Genomics* 22**,** 152-158.

Cha, P.C., Mushiroda, T., Takahashi, A., Kubo, M., Minami, S., Kamatani, N., and Nakamura, Y. (2010). Genome-wide association study identifies genetic determinants of warfarin responsiveness for Japanese. *Hum Mol Genet* 19**,** 4735-4744.

Chen, Y., Ferguson, S.S., Negishi, M., and Goldstein, J.A. (2004). Induction of human CYP2C9 by rifampicin, hyperforin, and phenobarbital is mediated by the pregnane X receptor. *J Pharmacol Exp Ther* 308**,** 495-501.

Ciccacci, C., Paolillo, N., Di Fusco, D., Novelli, G., and Borgiani, P. (2011). EPHX1 polymorphisms are not associated with warfarin response in an Italian population. *Clin Pharmacol Ther* 89**,** 791; author reply 792.

Crespi, C.L., and Miller, V.P. (1997). The R144C change in the CYP2C9*2 allele alters interaction of the cytochrome P450 with NADPH:cytochrome P450 oxidoreductase. *Pharmacogenetics* 7**,** 203-210.

D'ambrosio, R.L., D'andrea, G., Cappucci, F., Chetta, M., Di Perna, P., Brancaccio, V., Grandone, E., and Margaglione, M. (2004). Polymorphisms in factor II and factor VII genes modulate oral anticoagulation with warfarin. *Haematologica* 89**,** 1510-1516.

D'andrea, G., D'ambrosio, R.L., Di Perna, P., Chetta, M., Santacroce, R., Brancaccio, V., Grandone, E., and Margaglione, M. (2005). A polymorphism in the VKORC1 gene is associated with an interindividual variability in the dose-anticoagulant effect of warfarin. *Blood* 105**,** 645-649.

Dahlback, B. (2005). Blood coagulation and its regulation by anticoagulant pathways: genetic pathogenesis of bleeding and thrombotic diseases. *J Intern Med* 257**,** 209-223.

Daly, A.K. (2013). Optimal dosing of warfarin and other coumarin anticoagulants: the role of genetic polymorphisms. *Arch Toxicol* 87**,** 407-420.

European Bioinformatics Lnstitute (2018). "The International Genome Sample Resource (IGSR) Providing ongoing support for the 1000 Genomes Project data Human (Ensembl GRCh38) ".).

Haining, R.L., Hunter, A.P., Veronese, M.E., Trager, W.F., and Rettie, A.E. (1996). Allelic variants of human cytochrome P450 2C9: baculovirus-mediated expression, purification, structural characterization, substrate stereoselectivity, and prochiral selectivity of the wild-type and I359L mutant forms. *Arch Biochem Biophys* 333**,** 447-458.

Huang, S.W., Xiang, D.K., Huang, L., Chen, B.L., An, B.Q., Li, G.F., and Luo, Z.Y. (2011). Influence of GGCX genotype on warfarin dose requirements in Chinese patients. *Thromb Res* 127**,** 131-134.

Johnson, J.A., Caudle, K.E., Gong, L., Whirl-Carrillo, M., Stein, C.M., Scott, S.A., Lee, M.T., Gage, B.F., Kimmel, S.E., Perera, M.A., Anderson, J.L., Pirmohamed, M., Klein, T.E., Limdi, N.A., Cavallari, L.H., and Wadelius, M. (2017). Clinical Pharmacogenetics Implementation Consortium (CPIC) Guideline for Pharmacogenetics-Guided Warfarin Dosing: 2017 Update. *Clin Pharmacol Ther* 102**,** 397-404.

Johnson, J.A., and Cavallari, L.H. (2013). Pharmacogenetics and cardiovascular disease--implications for personalized medicine. *Pharmacol Rev* 65**,** 987-1009.

Johnson, J.A., and Cavallari, L.H. (2015). Warfarin pharmacogenetics. *Trends Cardiovasc Med* 25**,** 33-41.

Jorgensen, A.L., Fitzgerald, R.J., Oyee, J., Pirmohamed, M., and Williamson, P.R. (2012). Influence of CYP2C9 and VKORC1 on patient response to warfarin: a systematic review and meta-analysis. *PLoS One* 7**,** e44064.

Kamali, X., Wulasihan, M., Yang, Y.C., Lu, W.H., Liu, Z.Q., and He, P.Y. (2013). Association of GGCX gene polymorphism with warfarin dose in atrial fibrillation population in Xinjiang. *Lipids Health Dis* 12**,** 149.

Kimura, R., Miyashita, K., Kokubo, Y., Akaiwa, Y., Otsubo, R., Nagatsuka, K., Otsuki, T., Okayama, A., Minematsu, K., Naritomi, H., Honda, S., Tomoike, H., and Miyata, T. (2007). Genotypes of vitamin K epoxide reductase, gamma-glutamyl carboxylase, and cytochrome P450 2C9 as determinants of daily warfarin dose in Japanese patients. *Thromb Res* 120**,** 181-186.

Kroetz, D.L., Pauli-Magnus, C., Hodges, L.M., Huang, C.C., Kawamoto, M., Johns, S.J., Stryke, D., Ferrin, T.E., Deyoung, J., Taylor, T., Carlson, E.J., Herskowitz, I., Giacomini, K.M., Clark, A.G., and Pharmacogenetics of Membrane Transporters, I. (2003). Sequence diversity and haplotype structure in the human ABCB1 (MDR1, multidrug resistance transporter) gene. *Pharmacogenetics* 13**,** 481-494.

Liang, R., Wang, C., Zhao, H., Huang, J., Hu, D., and Sun, Y. (2012). Influence of CYP4F2 genotype on warfarin dose requirement-a systematic review and meta-analysis. *Thromb Res* 130**,** 38-44.

Loebstein, R., Vecsler, M., Kurnik, D., Austerweil, N., Gak, E., Halkin, H., and Almog, S. (2005). Common genetic variants of microsomal epoxide hydrolase affect warfarin dose requirements beyond the effect of cytochrome P450 2C9. *Clin Pharmacol Ther* 77**,** 365-372.

Mcdonald, M.G., Rieder, M.J., Nakano, M., Hsia, C.K., and Rettie, A.E. (2009). CYP4F2 is a vitamin K1 oxidase: An explanation for altered warfarin dose in carriers of the V433M variant. *Mol Pharmacol* 75**,** 1337-1346.

Mlynarsky, L., Bejarano-Achache, I., Muszkat, M., and Caraco, Y. (2012). Factor VII R353Q genetic polymorphism is associated with altered warfarin sensitivity among CYP2C9 *1/*1 carriers. *Eur J Clin Pharmacol* 68**,** 617-627.

Moon, J.Y., Chang, B.C., Lee, K.E., Bang, J.S., and Gwak, H.S. (2015). Effects of Pregnane X Receptor Genetic Polymorphisms on Stable Warfarin Doses. *J Cardiovasc Pharmacol Ther* 20**,** 532-538.

Nakagawa, T., Kishino, S., Itoh, S., Sugawara, M., and Miyazaki, K. (2003). Differential binding of disopyramide and warfarin enantiomers to human alpha(1)-acid glycoprotein variants. *Br J Clin Pharmacol* 56**,** 664-669.

National Center for Biotechnology Information (2018). "Resources".).

Pautas, E., Moreau, C., Gouin-Thibault, I., Golmard, J.L., Mahe, I., Legendre, C., Taillandier-Heriche, E., Durand-Gasselin, B., Houllier, A.M., Verrier, P., Beaune, P., Loriot, M.A., and Siguret, V. (2010). Genetic factors (VKORC1, CYP2C9, EPHX1, and CYP4F2) are predictor variables for warfarin response in very elderly, frail inpatients. *Clin Pharmacol Ther* 87**,** 57-64.

Perera, M.A., Cavallari, L.H., Limdi, N.A., Gamazon, E.R., Konkashbaev, A., Daneshjou, R., Pluzhnikov, A., Crawford, D.C., Wang, J., Liu, N., Tatonetti, N., Bourgeois, S., Takahashi, H., Bradford, Y., Burkley, B.M., Desnick, R.J., Halperin, J.L., Khalifa, S.I., Langaee, T.Y., Lubitz, S.A., Nutescu, E.A., Oetjens, M., Shahin, M.H., Patel, S.R., Sagreiya, H., Tector, M., Weck, K.E., Rieder, M.J., Scott, S.A., Wu, A.H., Burmester, J.K., Wadelius, M., Deloukas, P., Wagner, M.J., Mushiroda, T., Kubo, M., Roden, D.M., Cox, N.J., Altman, R.B., Klein, T.E., Nakamura, Y., and Johnson, J.A. (2013). Genetic variants associated with warfarin dose in African-American individuals: a genome-wide association study. *Lancet* 382**,** 790-796.

Ramirez, A.H., Shi, Y., Schildcrout, J.S., Delaney, J.T., Xu, H., Oetjens, M.T., Zuvich, R.L., Basford, M.A., Bowton, E., Jiang, M., Speltz, P., Zink, R., Cowan, J., Pulley, J.M., Ritchie, M.D., Masys, D.R., Roden, D.M., Crawford, D.C., and Denny, J.C. (2012). Predicting warfarin dosage in European-Americans and African-Americans using DNA samples linked to an electronic health record. *Pharmacogenomics* 13**,** 407-418.

Rettie, A.E., Wienkers, L.C., Gonzalez, F.J., Trager, W.F., and Korzekwa, K.R. (1994). Impaired (S)-warfarin metabolism catalysed by the R144C allelic variant of CYP2C9. *Pharmacogenetics* 4**,** 39-42.

Rieder, M.J., Reiner, A.P., Gage, B.F., Nickerson, D.A., Eby, C.S., Mcleod, H.L., Blough, D.K., Thummel, K.E., Veenstra, D.L., and Rettie, A.E. (2005). Effect of VKORC1 haplotypes on transcriptional regulation and warfarin dose. *N Engl J Med* 352**,** 2285-2293.

Ross, D., and Siegel, D. (2004). NAD(P)H:quinone oxidoreductase 1 (NQO1, DT-diaphorase), functions and pharmacogenetics. *Methods Enzymol* 382**,** 115-144.

Rost, S., Fregin, A., Koch, D., Compes, M., Muller, C.R., and Oldenburg, J. (2004). Compound heterozygous mutations in the gamma-glutamyl carboxylase gene cause combined deficiency of all vitamin K-dependent blood coagulation factors. *Br J Haematol* 126**,** 546-549.

Schelleman, H., Brensinger, C.M., Chen, J., Finkelman, B.S., Rieder, M.J., and Kimmel, S.E. (2010). New genetic variant that might improve warfarin dose prediction in African Americans. *Br J Clin Pharmacol* 70**,** 393-399.

Schelleman, H., Chen, Z., Kealey, C., Whitehead, A.S., Christie, J., Price, M., Brensinger, C.M., Newcomb, C.W., Thorn, C.F., Samaha, F.F., and Kimmel, S.E. (2007). Warfarin response and vitamin K epoxide reductase complex 1 in African Americans and Caucasians. *Clin Pharmacol Ther* 81**,** 742-747.

Shendre, A., Brown, T.M., Liu, N., Hill, C.E., Beasley, T.M., Nickerson, D.A., and Limdi, N.A. (2016). Race-Specific Influence of CYP4F2 on Dose and Risk of Hemorrhage Among Warfarin Users. *Pharmacotherapy* 36**,** 263-272.

Shikata, E., Ieiri, I., Ishiguro, S., Aono, H., Inoue, K., Koide, T., Ohgi, S., and Otsubo, K. (2004). Association of pharmacokinetic (CYP2C9) and pharmacodynamic (factors II, VII, IX, and X; proteins S and C; and gamma-glutamyl carboxylase) gene variants with warfarin sensitivity. *Blood* 103**,** 2630-2635.

Spek, C.A., Koster, T., Rosendaal, F.R., Bertina, R.M., and Reitsma, P.H. (1995). Genotypic variation in the promoter region of the protein C gene is associated with plasma protein C levels and thrombotic risk. *Arterioscler Thromb Vasc Biol* 15**,** 214-218.

Sullivan-Klose, T.H., Ghanayem, B.I., Bell, D.A., Zhang, Z.Y., Kaminsky, L.S., Shenfield, G.M., Miners, J.O., Birkett, D.J., and Goldstein, J.A. (1996). The role of the CYP2C9-Leu359 allelic variant in the tolbutamide polymorphism. *Pharmacogenetics* 6**,** 341-349.

Sussman, N., Waltershied, M., Butler, T., Cali, J., Riss, T., and Kelly, J. (2002). The predictive nature of high throughput toxicity screening using a human hepatocyte cell line. *Cell Notes* 3**,** 7-10.

Takeuchi, F., Mcginnis, R., Bourgeois, S., Barnes, C., Eriksson, N., Soranzo, N., Whittaker, P., Ranganath, V., Kumanduri, V., Mclaren, W., Holm, L., Lindh, J., Rane, A., Wadelius, M., and Deloukas, P. (2009). A genome-wide association study confirms VKORC1, CYP2C9, and CYP4F2 as principal genetic determinants of warfarin dose. *PLoS Genet* 5**,** e1000433.

Voora, D., Koboldt, D.C., King, C.R., Lenzini, P.A., Eby, C.S., Porche-Sorbet, R., Deych, E., Crankshaw, M., Milligan, P.E., Mcleod, H.L., Patel, S.R., Cavallari, L.H., Ridker, P.M., Grice, G.R., Miller, R.D., and Gage, B.F. (2010). A polymorphism in the VKORC1 regulator calumenin predicts higher warfarin dose requirements in African Americans. *Clin Pharmacol Ther* 87**,** 445-451.

Wadelius, M., Chen, L.Y., Downes, K., Ghori, J., Hunt, S., Eriksson, N., Wallerman, O., Melhus, H., Wadelius, C., Bentley, D., and Deloukas, P. (2005). Common VKORC1 and GGCX polymorphisms associated with warfarin dose. *Pharmacogenomics J* 5**,** 262-270.

Wadelius, M., Chen, L.Y., Eriksson, N., Bumpstead, S., Ghori, J., Wadelius, C., Bentley, D., Mcginnis, R., and Deloukas, P. (2007). Association of warfarin dose with genes involved in its action and metabolism. *Hum Genet* 121**,** 23-34.

Wadelius, M., and Pirmohamed, M. (2007). Pharmacogenetics of warfarin: current status and future challenges. *Pharmacogenomics J* 7**,** 99-111.

Wallin, R., and Hutson, S. (1982). Vitamin K-dependent carboxylation. Evidence that at least two microsomal dehydrogenases reduce vitamin K1 to support carboxylation. *J Biol Chem* 257**,** 1583-1586.

Yu, W.Y., Sun, X., Wadelius, M., Huang, L., Peng, C., Ma, W.L., and Yang, G.P. (2016). Influence of APOE Gene Polymorphism on Interindividual and Interethnic Warfarin Dosage Requirement: A Systematic Review and Meta-Analysis. *Cardiovasc Ther* 34**,** 297-307.

Yuan, H.Y., Chen, J.J., Lee, M.T., Wung, J.C., Chen, Y.F., Charng, M.J., Lu, M.J., Hung, C.R., Wei, C.Y., Chen, C.H., Wu, J.Y., and Chen, Y.T. (2005). A novel functional VKORC1 promoter polymorphism is associated with inter-individual and inter-ethnic differences in warfarin sensitivity. *Hum Mol Genet* 14**,** 1745-1751.
